# Comparative profiling of single-cell transcriptome reveals heterogeneity of tumor microenvironment between solid and acinar lung adenocarcinoma

**DOI:** 10.1186/s12967-022-03620-3

**Published:** 2022-09-23

**Authors:** Dianke Li, Huansha Yu, Junjie Hu, Shaoling Li, Yilv Yan, Shuangyi Li, Liangdong Sun, Gening Jiang, Likun Hou, Lele Zhang, Peng Zhang

**Affiliations:** 1 Department of Thoracic Surgery, Shanghai Pulmonary Hospital, Tongji University School of Medicine, No. 507 Zhengmin Road, Shanghai, 200433 China; 2 Experimental Animal Center, Shanghai Pulmonary Hospital, Tongji University School of Medicine, Shanghai, 200433 China; 3 Department of Pathology, Shanghai Pulmonary Hospital, Tongji University School of Medicine, No. 507 Zhengmin Road, Shanghai, 200433 China; 4 Central Laboratory, Shanghai Pulmonary Hospital, Tongji University School of Medicine, No. 507 Zhengmin Road, Shanghai, 200433 China; 5 Department of Thoracic Surgery, The First Affiliated Hospital of Shihezi University Medical College, Shihezi, 832000 Xinjiang China

**Keywords:** Lung adenocarcinoma, Histology, Tumor immune microenvironment, Tumor metabolism, Immunotherapy

## Abstract

**Background:**

The diversity of histologic composition reflects the inter- and intra-tumor heterogeneity of lung adenocarcinomas (LUADs) macroscopically. Insights into the oncological characteristics and tumor microenvironment (TME) of different histologic subtypes of LUAD at the single-cell level can help identify potential therapeutic vulnerabilities and combinational approaches to improve the survival of LUAD patients.

**Methods:**

Through comparative profiling of cell communities defined by scRNA-seq data, we characterized the TME of LUAD samples of distinct histologic subtypes, with relevant results further confirmed in multiple bulk transcriptomic, proteomic datasets and an independent immunohistochemical validation cohort.

**Results:**

We find that the hypoxic and acidic situation is the worst in the TME of solid LUADs compared to other histologic subtypes. Besides, the tumor metabolic preferences vary across histologic subtypes and may correspondingly impinge on the metabolism and function of immune cells. Remarkably, tumor cells from solid LUADs upregulate energy and substance metabolic activities, particularly the folate-mediated one-carbon metabolism and the key gene MTHFD2, which could serve as a potential therapeutic target. Additionally, ubiquitination modifications may also be involved in the progression of histologic patterns. Immunologically, solid LUADs are characterized by a predominance of exhausted T cells and immunosuppressive myeloid cells, where the hypoxic, acidified and nutrient-deprived TME has a non-negligible impact. Discrepancies in stromal cell function, evidenced by varying degrees of stromal remodeling and fibrosis, may also contribute to the specific immune phenotype of solid LUADs.

**Conclusions:**

Overall, our research proposes several potential entry points to improve the immunosuppressive TME of solid LUADs, thereby synergistically potentiating their immunotherapeutic efficacy, and may provide precise therapeutic strategies for LUAD patients of distinct histologic subtype constitution.

**Supplementary Information:**

The online version contains supplementary material available at 10.1186/s12967-022-03620-3.

## Background

Invasive lung adenocarcinomas (LUADs) account for almost 70–90% of all surgically resected lung cancers [1]. The morphologic manifestations of invasive LUADs have been well characterized microscopically and are mainly differentiated into lepidic, papillary, acinar, micropapillary, and solid growth patterns [1, 2]. The diversity of histologic composition macroscopically reflects the inter-tumor and intra-tumor heterogeneity of LUADs, with most LUADs manifesting as a successive tissue transition between two or more histologic patterns [2]. Solid and acinar are two histologic subtypes of LUAD with high frequency, the solid type was identified as a histologic pattern stronger in aggressiveness, higher in grade, and worse in prognosis than the acinar type [2]. Identification of patients who may benefit from additional treatment after curative surgery for early LUAD has been a focus in the field of adjuvant therapy. An early study suggested that the solid type benefited from adjuvant chemotherapy in terms of disease-free survival (DFS) and specific DFS, while the acinar type did not [3]. Moreover, the latest grading system, introduced with 20% or more of high-grade patterns (including the solid pattern) as the cut-off for histologically high-grade LUADs, consistently demonstrated that those patients with high-grade LUADs could benefit from adjuvant chemotherapy [4, 5].

To our knowledge, no study as yet has conclusively reported any association between the histologic subtype and the response to targeted or immune therapies, both in the adjuvant and neoadjuvant scenarios (comprehensive histological assessment relies on radical surgical excision). Although the application of targeted therapies depends on driver gene detection, it has been shown that the solid type has a lower frequency of targetable alterations when compared to the acinar type, as evidenced by rare EGFR mutations and a higher frequency of KRAS mutations, which implicates that tyrosine kinase inhibitors (TKIs) may not be satisfactorily effective in solid-type tumors [6, 7]. More importantly, several studies have proposed that the solid type may benefit from additional immunotherapy, especially the perioperative immune checkpoint blocker (ICB) treatment, which is supported by its higher TMB and PD-L1 expression [6–8]. Nevertheless, the efficacy of immunotherapy can be hampered by various factors, including hypoxic, acidic conditions and tumor metabolic reprogramming, which is precisely what makes the prospects for combinational treatment so attractive [9]. Whether the histologic constitution of LUAD serves to guide the determination of future combinational therapeutic regimens is largely unknown.

Since previous research addressing the molecular underpinnings of histologic subtypes was mostly based on bulk tumor sequencing [6, 7, 10, 11]. In-depth comprehension of the oncological characteristics as well as the tumor microenvironment of different histologic subtypes of LUAD at the single-cell level can help identify potential therapeutic vulnerabilities and combinational approaches to improve the survival of LUAD patients. Here we performed scRNA-seq analysis on solid-, and acinar-prevalent LUAD tumors, and adjacent normal lung samples. Through the comparative profiling of scRNA-seq data-defined cell communities in the tumor microenvironment (TME), we brought some new insights into the molding of tumor cells to their surrounding circumstances and the metabolic preferences of tumor cells from distinct histologic subtypes. Relevant results were further validated in multiple bulk transcriptomic and proteomic datasets as well as in an independent immunohistochemical validation cohort. Moreover, the constitutional and functional differences of immune and stromal cell subpopulations were also addressed. Our study promises to provide some attractive clues and supporting evidences for histologic subtype-oriented therapy in patients with LUADs.

## Methods

### Data resources

The scRNA-seq data of untreated, primary LUAD samples were collected from two of our previously published researches [12, 13]. The histologic constituents of each tumor were re-accessed and recorded semi-quantitatively by two experienced pathologists (L.H. and S.L.) as suggested by the World Health Organization [2]. The clinicopathological information of the enrolled samples was summarized in Additional file 1: Table S1. For the complementation of scRNA-seq data, three additional LUAD cohorts comprising mRNA or protein expression datasets were also collected. The RNA-seq data for The Cancer Genome Atlas (TCGA) cohort was downloaded from UCSC Xena (http://xena.ucsc.edu/) and the corresponding histopathologic information was obtained from the work presented by The Cancer Genome Atlas Research Network [14]. The RNA-seq data and histopathological information for the East Asian ancestry (EAS) cohort were derived from Chen et al.'s work [15] and obtained from OncoSG (https://src.gisapps.org/OncoSG/)(16, 17). The RNA-seq and proteomic data, along with histopathological information for the Clinical Proteomic Tumor Analysis Consortium (CPTAC) cohort were obtained from Gillette et al.’s research [18]. The histologic categories of these LUAD samples were determined by their predominant histologic pattern. In addition, for exploring the correlations between tumor cell metabolic pathway activities and the ratio of exhausted CD8 + T cells, an independent LUAD scRNA-seq dataset was used [19], and the processed data (including the normalized log2TPM matrix together with the cell annotation table) was downloaded from the Gene Expression Omnibus database (Accession Code: GSE131907).

We also retrospectively collected an additional cohort from our center which included 16 patients with untreated, surgically resected LUADs, and the formalin-fixed paraffin-embedded (FFPE) samples from each patient were obtained. The basic clinical and histologic information for this validation cohort was provided in Additional file 1: Table S1. The study was conducted following the principles of the Declaration of Helsinki, and the study protocol was approved by the ethics committee of Shanghai Pulmonary Hospital. Because of the retrospective nature of the study, patient consent for inclusion was waived.

### scRNA-seq data processing and analysis

For all enrolled samples with scRNA-seq data, the specific procedures for single cell suspension preparation, library construction and sequencing were detailed in the original article [12, 13]. Raw sequence files in fastq format were obtained and processed with Cell Ranger (version 4.0.0) coupled with the GRCh38 human reference genome to generate a gene count matrix for each sample. Then Seurat R package took over the downstream analytic procedures [20, 21]. First of all, cells met any following criteria were removed: (1) Cells with extreme feature counts (< 500 or > 6000); (2) Cells with extreme RNA counts (> 40,000); (3) > 10% reads aligned to mitochondria; (4) < 10% reads aligned to ribosome. Subsequently, we performed data normalization, identification of highly variable genes and principal component analysis (PCA) with Seurat’s classic workflow. Then, harmony algorithm was used to correct the potential batches among sample resources. Uniform Manifold Approximation and Projection (UMAP) was facilitated for dimension reduction with the parameter “reduction” set as “harmony”. The Seurat “FindNeighbors” and “FindClusters” functions were applied to detect communities and find cell clusters. And then the “FindAllMarkers” function with default settings was used to find markers for each identified cluster.

### Cell annotation and doublets removal

All cells were preliminarily labeled as epithelium, immune and stromal cells (roughly, PTPRC for marking immune cells, EPCAM for epitheliums, VWF and COL1A2 for stromal cells). Annotation of primary immune cell types was implemented according to the expression of canonical cell markers and by inspecting the top marker genes of each cluster. Notably, cell clusters expressing two or more major cell lineage markers were manually removed due to their potential doublet identity (such as the co-expression of LYZ for myeloid cells and CD3E for T cells in one single cluster).

### CNV estimation

Large-scale chromosomal copy number variations within the cancer cells at single-cell level were identified by inferCNV (https://github.com/broadinstitute/inferCNV) with stromal cells (fibroblasts and endothelium) as normal reference. The Hidden Markov Model (HMM) model was used to infer the variation degrees by setting “HMM = TRUE, denoise = TRUE”.

### Differential expression analysis and gene set variation analysis (GSVA)

The Seurat “FindMarker” function was used to identify differentially expressed genes between cells from solid and acinar samples under the default Wilcoxon rank-sum test. To estimate pathway activity at single-cell level, we applied GSVA with default parameters, as implemented in the GSVA R package (version1.32.0), as previously described [22]. The gene sets of hallmarks (h.all.v7.2.symbols.gmt) and KEGG pathways (c2.cp.kegg.v7.2.symbols.gmt) used in this study were acquired from the GSEA website (https://www.gsea-msigdb.org/gsea/index.jsp) [23, 24]. The limma R package (version 3.42.2) was used to calculate the differential activities of pathways between groups. A Benjamini-Hochberg-corrected P value of ≤ 0.05 was used to identify significantly altered pathways.

### Scoring of gene signatures in scRNA-seq

For exploring the functional state of immune cells from tumors of different histologic types, we curated gene expression signatures including the cytotoxic, exhausted and hypoxic signatures for T/NK cells [25]; the M1 and M2 phenotype signatures for macrophages [26]; the antigen presentation and immunosuppressive signatures for DCs [27]; the fibrillar collagens and epithelial mesenchymal transition signatures for fibroblasts [28]. The detailed list of genes for each signature and their reference sources were provided in the corresponding supplemental tables. The normalized weighted mean expression of those signatures was calculated by Seurat’s “AddModuleScore” function, and signatures expression among subgroups was compared by two-sided Wilcoxon rank-sum test.

### Cellular fraction calculation

To compare the variation in the percentage composition of immune cell subpopulations between different histologic subgroups, we calculated the fraction of cell numbers of the resulting cell subpopulations to the total number of clustered cells at the individual sample level. The significance of differences among histologic subgroups for the fractions was compared using two-sided unpaired Wilcoxon rank-sum test.

### Analyses on the GEPIA2 web server

The prognostic relevance of molecular expression in the TCGA LUAD cohort was analyzed on the GEPIA2 (http://gepia2.cancer-pku.cn/) web server [29]. Specifically, the “Survival Analysis” module was selected. Patient groups were divided by the median of molecular expression, the Kaplan–Meier curves were plotted to visualize survival differences, the cox proportional hazard ratio (HR) was calculated and Log-rank test was used for hypothesis test. Additionally, correlation analysis between the expression of MTHFD2 and UBE2S in the TCGA LUAD cohort was performed on the “Correlation Analysis” module by Pearson correlation test.

### Molecular expression in bulk transcriptome and proteomic datasets

Differential expression analyses of molecules of interest among histologic types were performed on three RNA-seq datasets (log2-transformed FPKM expression values for TCGA and EAS cohorts, and log2-transformed RPKM for CPTAC cohort), and the CPTAC proteomic dataset (Two-component-normalized log2-transformed protein expression values). Pairwise comparisons of the solid type and other histological types were performed by two-sided Wilcoxon rank-sum tests.

### Gene signatures enrichment in bulk RNA-seq

The enrichment analyses of gene signatures in the TCGA bulk RNA-seq dataset were performed by gsva function in the GSVA R package (version 1.42.0) with parameters “method = ssgsea, kcdf = Gaussian, abs.ranking = TRUE”. The detailed list of genes for each signature and their reference sources are provided in Additional file 2: Table S2. Comparisons of signature enrichment scores across histologic subtypes were performed using the Kruskal–Wallis test. Violin plots were drawn by the ggpubr R package (version 0.4.0) for visualization.

### Metabolic pathway analysis

For comprehensive dissecting the metabolic differences of epitheliums across histologic subgroups in our scRNA-seq dataset, we quantified the metabolic activities of every single epithelium by applying the scMetabolism R tool pipeline with parameters “method = VISION, metabolism.type = KEGG” [30, 31]. The median absolute deviation value was then used to measure the degree of inter-subgroup variation in metabolic pathway activity, only the top 50% variable metabolic pathways were selected for heatmap plotting.

For the independent LUAD scRNA-seq dataset, only samples from tumor lung (tLung) were included [19]. We also removed two samples with less than 50 tumor cells, and nine samples remained for further analysis. CD8 + T cells in the dataset were annotated as CD8 low T, Cytotoxic CD8 + T, Exhausted CD8 + T and Naive CD8 + T, as described in the original manuscript [19]. The exhausted CD8 + T cell ratio of each sample was calculated. We next quantified the metabolic activities of every single tumor cell in the same manner as described above. For each sample, the score on a specific metabolic pathway was calculated as the average score of tumor cells in that sample on that metabolic pathway. Finally, Pearson’s correlation tests were applied to examine the correlations between exhausted CD8 + T cell ratio and tumor metabolic pathway activities, with correction for multiple testing by Benjamini–Hochberg method, only metabolic pathways with P values less than 0.1 were selected for heatmap mapping.

### Immunohistochemistry

Tissues were fixed in 4% paraformaldehyde, embedded in paraffin, cut into sections, and placed on adhesion microscope slides. Sections were subjected to immunohistochemical (IHC) staining according to standard procedures. We performed the IHC by using the MTHFD2 mouse anti-human antibody (Abcam, ab56772). The primary antibody was incubated at 4 °C overnight followed by using the BOND™ Polymer Refine Detection Kit (Leica, DS9800) according to the manufacturer’s instructions. Whole slide scanning was performed using panoramic MIDI under a 40 × objective lens. For each slide, the histologic patterns were firstly identified according to the cellular structure of the tumor, then three to five non-overlapping fields of view for each histologic region were randomly captured at 100 × magnification, and the staining intensity of MTHFD2 was finally semi-quantified using the Image J software (1.53q) by transforming it into mean optical density [32]. The statistical difference in staining intensity of MTHFD2 between solid and lepidic/acinar was determined by the Wilcoxon rank-sum test.

### Statistics

The statistical analyses involved in this study were described in the corresponding method section. All statistical analyses and data presentations were performed by the R program (versions 3.6.3 and 4.0.2). All reported P values were two-tailed, and P < 0.05 was considered statistically significant.

## Results

### Analysis of scRNA-seq data from histologically annotated LUAD samples

The present study was a repurposing of scRNA-seq data from two of our previously published researches [12, 13]. All surgically excised samples came from patients with untreated, primary non-metastatic LUADs. The histologic constituents of each tumor sample were assessed and recorded semi-quantitatively [1, 2]. We attempted to single out tumor samples with high histologic purity for the purpose of dissecting subtype-specific oncological and immunological characteristics. Collectively, four solid-type, four acinar-type LUAD samples, and five adjacent normal lung samples were enrolled in this study. The representative hematoxylin–eosin (HE) stained images clearly visualized the microscopic structure of the acinar and solid patterns (Additional file 7: Fig. S1A–B). There was a "near-pure" tumor with the solid pattern covering more than 70% of the whole tumor in each solid LUAD sample [33]. With regards to the acinar type, the proportion of acinar pattern in each sample was greater than 50%, with the content of solid/micropapillary patterns limited to less than 10%, allowing to minimize the impacts of high-grade histologic components. The clinicopathological information for all enrolled samples was summarized in Additional file 1: Table S1.

The single-cell transcriptomic profiles generated by each sample were then combined for integrated analysis. Following strict quality control procedures, a sparse matrix with 97,875 cells and 25,233 genes was obtained (Methods). Before performing unsupervised graph-based clustering analysis, potential batch effects between samples were assessed and eliminated. Subsequently, all cells were labeled preliminary based on the expression of canonical cell markers (roughly, PTPRC for immune cells, EPCAM for epitheliums, VWF and COL1A2 for stromal cells; Additional file 7: Fig. S1C-D). Among these cells, 30,208 (30.86%) originated from solid samples, 25,250 (25.80%) originated from acinar samples, and 42,417 (43.34%) originated from adjacent lung tissues.

### Tumor cells from solid LUADs create a more anoxic and acidic TME

**Fig. 1:**
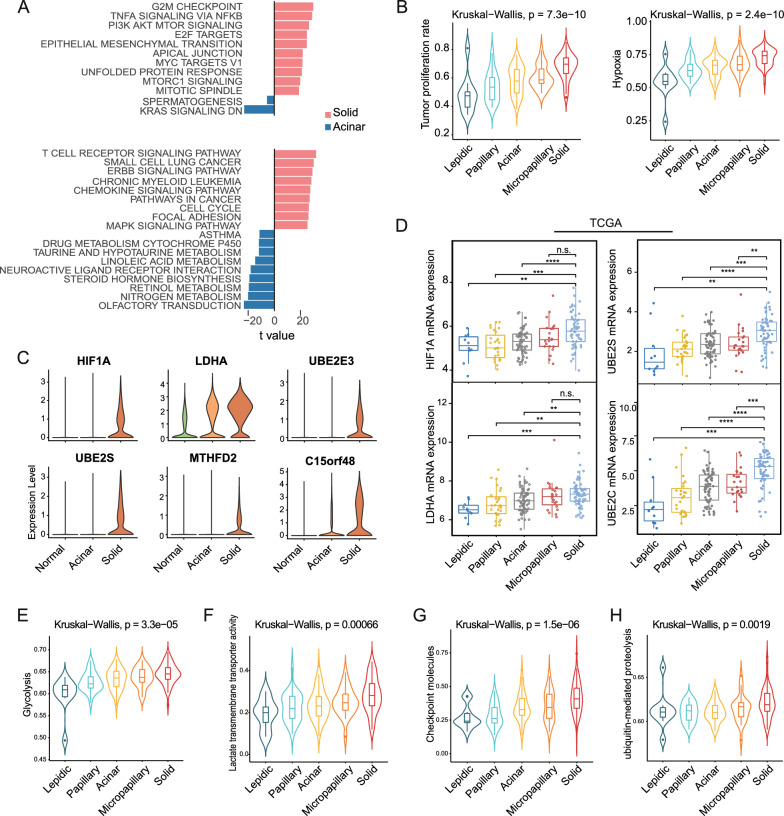
Tumor cells from solid LUADs create a more anoxic and acidic tumor microenvironment. **A**. Differentially enriched hallmarks (top) and KEGG pathways (bottom) between tumor cells from solid and acinar samples revealed by GSVA. **B**. Violin plots showing enrichment scores of tumor proliferating rate and hypoxia signatures by histologic subtypes in the TCGA LUAD cohort. Global differences were measured by the Kruskal–Wallis test. **C**. Violin plots of upregulated genes in solid LUAD tumor cells. **D**. Boxplots showing mRNA expression of HIF1A, LDHA, UBE2S and UBE2C by histologic subtypes in the TCGA LUAD cohort. Box centerlines, median; box limits, the 25th and 75th percentiles; box whiskers, 1.5 × the interquartile range. Comparisons were performed using two-sided Wilcoxon rank-sum test (*P < 0.05, **P < 0.01, ***P < 0.001, ****P < 0.0001, *n s* not significant). **E–H.** Violin plots showing enrichment scores of glycolysis **(E)**, lactate transmembrane transporter activity **(F)**, checkpoint molecules **(G)** and ubiquitin mediated proteolysis **(H)** signatures by histologic subtypes in the TCGA LUAD cohort. Global differences were measured by the Kruskal–Wallis test

We then committed to comparing the transcriptional characteristics of tumor cells derived from solid or acinar samples. By inferring large-scale copy number variations from transcriptome information, extensive chromosomal aberrations were observed in tumor-derived epitheliums relative to stromal cells (Additional file 7: Fig S1E). Comparing solid and acinar samples using gene set variation analyses (GSVA) [34] revealed that hallmarks associated with aggressiveness and metabolic activity, such as G2M checkpoint, angiogenesis, epithelial-mesenchymal transition (EMT), MYC targets V1 and PI3K/AKT/mTOR signaling, were up-regulated in tumor cells from solid samples (Fig. 1A, Additional file 2: Table S2), which was consistent with a more aggressive histopathological phenotype of solid LUADs. Notably, immune response-related hallmarks (such as TNFα signaling via NF-κB, IL2-STAT5 signaling, and IL6-JAK-STAT3 signaling) were also significantly enriched in solid samples. These findings emphasized the invasiveness of tumor cells from solid LUADs as well as their adept immune evasion capabilities.

It has been well established that hypoxia and acidification characterized the tumor microenvironment [35]. When comparing tumor cells from solid and acinar samples, hypoxia and glycolysis hallmarks were found to be more prominent in the former (Additional file 2: Table S2). Consistently, by applying single sample enrichment analysis (ssGSEA) in bulk RNA-seq data from the TCGA LUAD cohort, we found the enrichment scores of the tumor proliferating rate [36] and hypoxia [25] signatures were increased stepwise with histologic progression (Fig. 1B; Additional file 2: Table S2). Moreover, the expression levels of hypoxia-inducible factor-1 alpha (HIF1A) and lactate dehydrogenase A (LDHA), were observably upregulated in tumor cells from solid LUADs (Fig. 1C, Additional file 2: Table S2). As the key mediator of hypoxic response, HIF1A was intimately linked to multiple aspects of antitumor immunobiological processes [37]. And LDHA was identified as a transcription target of the oncogene MYC and was required for enhanced glycolysis and malignant potential of tumor cells [38]. LDHA was also an essential enzyme in lactate production, which was reported to create an acidic tumor microenvironment and mediate immunotolerance [35]. The upregulation of HIF1A and LDHA in solid LUADs was further corroborated in three publicly available bulk RNA-seq cohorts for which the histologic annotation data were available (Fig. 1D, Additional file 8: Fig. S2A, B; Methods). Moreover, enhanced glycolysis and lactate transmembrane transporter activity were also observed in solid LUADs in the TCGA cohort (Fig. 1E, F; Additional file 2: Table S2). These findings supported the hypothesis that tumor cells of solid LUADs constructed a more anoxic and acidic peritumor niche, which not only facilitated malignant progression but also impaired antitumor effector cell function. Indeed, immune suppression by checkpoint molecules was found to be most intense in solid LUADs (Fig. 1G; Additional file 2: Table S2).

### The role of epigenetic mechanisms in the differentiation of LUAD morphological subtypes

Previous research established the critical role of epigenetic mechanisms in determining the histologic identity of LUAD cells [10]. Intriguingly, we noticed the activity of the ubiquitin-mediated proteolysis pathway, as well as the expression of multiple ubiquitin-conjugating enzymes including UBE2S, UBE2E3 and UBE2C, were upregulated in tumor cells from solid samples (Fig. 1C; Additional file 2: Table S2). A consistent trend in the activity of the ubiquitin-mediated proteolysis across histologic subtypes was also observed in the TCGA cohort (Fig. 1H; Additional file 2: Table S2). Indeed, ubiquitin-conjugating enzymes have been shown to be overexpressed in various cancer types and involved in the regulation of a variety of cancer-associated biological processes [39]. Overexpression of UBE2S, for instance, has been shown to reduce the stability and activity of the p53 protein by increasing its ubiquitination [40]. Furthermore, UBE2S was demonstrated to be negatively correlated with the von Hippel-Lindau tumor-suppressor (pVHL), which mediated the ubiquitin-dependent proteolysis of HIF1A, in multiple tumor cell lines [41, 42]. Actually, ubiquitination engaged in metabolic reprogramming of cancer cells by modifying metabolic signaling pathways, transcription factors and metabolic enzymes [43]. The solid LUAD-enriched PI3K/AKT/mTOR and c-Myc signaling, could be enhanced by ubiquitination modifications in tumor cells [43]. Importantly, the differential expression of UBE2S and UBE2C across histologic subtypes were further corroborated in bulk transcriptome from the TCGA and the East Asian ancestry (EAS) cohorts, as well as proteome from the Clinical Proteomic Tumor Analysis Consortium (CPTAC) cohort (Fig. 1D, Additional file 8: Fig. S2C–D; Methods). Briefly, our results hinted that post-transcriptional modification represented by ubiquitination might play a crucial part in the acquisition of morphological phenotypes and the development of adaptive metabolic reprogramming in LUADs.

### Metabolic preferences vary between histologic subtypes

**Fig. 2:**
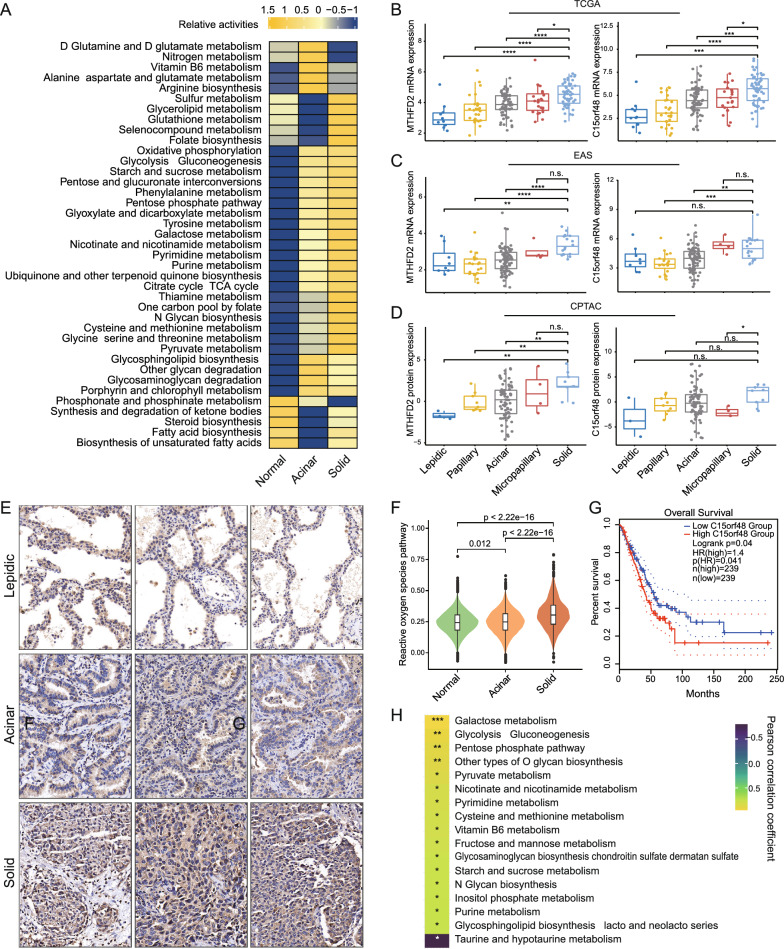
Metabolic preferences vary between histologic subtypes. **A**. Heatmap showing relative metabolic pathways activities in the three histologic subgroups. **B-D**. Boxplots showing MTHFD2 and C15orf48 mRNA expression in the TCGA **(B)** and the EAS cohorts **(C)**, and protein expression in the CPTAC cohort **(D)**. Box centerlines, median; box limits, the 25th and 75th percentiles; box whiskers, 1.5 × the interquartile range. Comparisons were performed using two-sided Wilcoxon rank-sum test (*P < 0.05, **P < 0.01, ***P < 0.001, ****P < 0.0001, *n s* not significant). **E**. Representative immunohistochemical images of MTHFD2 in lepidic, acinar and solid tumor regions. Scale bar, 50 μm. **F**. Violin plots showing the reactive oxygen species pathway score across the three histologic subgroups. Comparisons were performed by two-sided Wilcoxon rank-sum test. **G**. Kaplan–Meier survival curves showing the prognostic difference between the low and high C15orf48 expression groups in the TCGA LUAD cohort. **H**. Correlations between tumor metabolic pathway scores and the ratio of exhausted CD8 + T cells. P values were determined by Pearson's correlation test (*P < 0.1, **P < 0.05, ***P < 0.01)

We noticed a striking difference in the enrichment of multiple metabolic pathways between tumor cells derived from solid and acinar samples (Fig. 1A, Additional file 2: Table S2). Tumor cells in solid samples, for instance, had enhanced glycolysis and oxidative phosphorylation, while tumor cells from acinar samples had increased nitrogen metabolism. We next quantified a comprehensive collection of metabolic pathways [30] to dissect the metabolic landscape of epitheliums across groups (Fig. 2A). Remarkably, activities of some metabolic pathways, such as glycolysis/gluconeogenesis, purine and pyrimidine metabolism, and the citrate cycle (TCA cycle), showed progressive increases in lung epitheliums/tumor cells from normal to acinar and then solid samples. Nevertheless, some metabolic pathways exhibited histologic pattern preference. Specifically, tumor cells from solid samples were distinguished by increased activity in sulfur, glycerolipid, glutathione, selenocompound metabolism, and folate biosynthesis, whereas tumor cells from acinar samples were recognized by enhanced activity in D-glutamine and D-glutamate, nitrogen, vitamin B6 metabolism, and arginine biosynthesis. From a global perspective, tumor cells from solid LUADs upregulated pathways in both energy and substance metabolism (including nucleotide, amino acid, lipid metabolism and TCA cycle), which corresponds to their requirement for robust growth, proliferation and invasion. Among all these pathways, folate-mediated one-carbon metabolism supports a range of anabolic processes essential for cancer cell survival and growth [44]. Importantly, we discovered that the methylenetetrahydrofolate dehydrogenase 2 (MTHFD2), a key enzyme in the transformation of folate metabolites, was markedly upregulated in tumor cells from solid LUADs, along with the enhancement of folate biosynthesis and one-carbon pool by folate (Fig. 1C, Fig. 2A; Additional file 2: Table S2). Encouragingly, the expression differences of MTHFD2 across histologic subtypes were further confirmed in the TCGA and EAS bulk transcriptome cohorts, as well as the CPTAC proteomic cohort (Fig. 2B–D). More importantly, immunohistochemical staining further verified at the clinical sample scale that the density of MTHFD2 was increased in tumor regions of the solid pattern compared to the lepidic/acinar pattern (Fig. 2E, Additional file 9: Fig. S3A). To our knowledge, overexpression of MTHFD2 promotes tumorigenesis and malignant progression of various malignancies by virtue of its dual functions in metabolism and epigenetic modification [44–46]. The overexpression of MTHFD2 was associated with a worse prognosis in the TCGA LUAD cohort (Additional file 9: Fig. S3B). Strikingly, MTHFD2 raises the basal and IFN-γ-stimulated PD-L1 expression in both basal and IFN-γ-stimulating conditions, which could be the epitome of the linking of onco-metabolic genes and tumor immunoresistance [45]. Intriguingly, a strong positive correlation between the expression of MTHFD2 and UBE2S was observed in the TCGA cohort (Additional file 9: Fig. S3C), suggesting a possible link between ubiquitination modifications and metabolic reprogramming in LUADs.

What’s more, we noticed the enhancement of glutathione metabolism and the enrichment of reactive oxygen species (ROS) pathway in tumor cells from solid samples (Fig. 2A, F; Additional file 2: Table S2). This suggested that tumor cells in the solid type might confront a more intense oxidative stress reaction. Consistently, the apoptosis hallmark was significantly enriched in tumor cells from solid LUADs (Additional file 2: Table S2). ROS are a complex class of molecules with both pro-tumor and anti-tumor effects [47]. Excessive ROS can induce cell death via various approaches, such as apoptosis, necrosis and autophagy, thus limiting the progression of cancer [48]. Interestingly, we discovered that the gene C15orf48 (also known as modulator of cytochrome C oxidase during Inflammation; MOCCI), which may be involved in the production of reactive oxygen species, was significantly upregulated in tumor cells from solid LUADs (Fig. 1C). C15orf48 is located in the cytochrome C oxidase subunit (Complex IV) in mitochondria and has been reported to reduce mitochondrial membrane potential and ROS production during inflammation, resulting in cellular protection and attenuated immunoreaction [49]. The expression differences of C15orf48 among LUAD subtypes were verified in the TCGA and EAS bulk transcriptome cohorts, as well as the CPTAC proteomic cohort (Fig. 2B–D). Survival analysis further demonstrated that high expression of C15orf48 was associated with a poor prognosis in LUAD patients in the TCGA cohort (Fig. 2G).

We wondered whether subtype metabolic preferences affect antitumor immune function. Using a publicly available scRNA-seq dataset of LUAD patients [19], we investigated the correlations between cancer cell metabolic pathway activity and the ratio of exhausted CD8 + T cells (Fig. 2H, Methods). The results showed unequivocally that the activation of metabolic pathways such as galactose metabolism, glycolysis/gluconeogenesis, and pentose phosphate pathway, was positively correlated with the ratio of exhausted CD8 + T cells. Most of these pathways were upregulated in tumor cells from solid samples except for vitamin B6 metabolism, which was relatively more active in acinar samples. Remarkably, through the Leloir pathway, galactose can be metabolized to produce G6P as an alternative fuel for glycolysis [50]. For the complementary of scRNA-seq, we quantified the enrichment scores of one-carbon pool by folate, pyrimidine metabolism and galactose metabolism in the TCGA LUAD cohort with bulk RNA-seq data (Additional file 9: Fig. S3D–F; Additional file 2: Table S2). The results showed that these metabolic pathways exhibited cascade enhancement with histological progression and reached their highest in the solid subtype, which is consistent with our previous findings.

In summary, the metabolic preferences of different histologic subtypes were consistent with their corresponding biological behavioral traits, especially the upregulation of folate-mediated one-carbon metabolism and the key gene MTHFD2 by tumor cells of solid LUADs to satisfy their cellular replication and transcriptional requirements. More importantly, we also gained some observations on the influence of tumor metabolic preferences on anti-tumor immune responses, which might provide new insights for understanding the heterogeneity of LUAD subtypes and exploring combinatorial therapeutic strategies.

### Compromised anti-tumor effects in T cells from solid samples

**Fig. 3:**
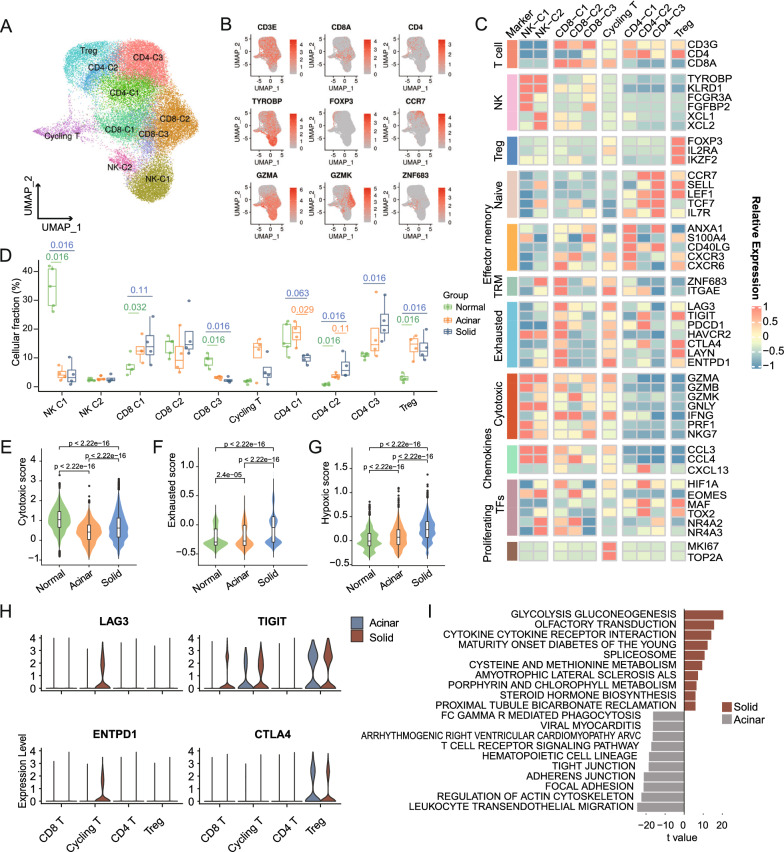
Compromised anti-tumor effects in solid LUAD-derived T cells. **A**. Cluster-colored UMAP plot of T/NK cells. **B**. UMAP plots showing the expression of chosen canonical T/NK cell marker genes. **C**. Heatmap depicting the normalized expression of canonical T/NK cell marker genes among clusters. **D**. Boxplot showing cellular fractions of each T/NK cluster in normal (n = 5), acinar (n = 4) and solid (n = 4) samples. Box centerlines, median; box limits, the 25th and 75th percentiles; box whiskers, 1.5 × the interquartile range. Line segments of different lengths indicate which two groups were compared. Two-sided Wilcoxon rank-sum test. **E–G**. Violin and box plots showing the cytotoxic **(E)**, exhausted **(F)** and hypoxic **(G)** signature scores for CD8 + T cells in the three subgroups. Comparisons were performed by two-sided Wilcoxon rank-sum test. **H**. Violin plots showing immune checkpoints expression of T cells from solid and acinar samples. **I**. Differentially enriched KEGG pathways between CD8 + T cells from solid and acinar samples revealed by GSVA

We next investigated the landscape of the tumor immune microenvironment across distinct histologic subtypes. Immune cells (n = 81,136) were re-clustered and identified as T cells, NK cells, myeloid cells, mast cells, plasmacytoid dendritic cells (pDCs), B cells, and plasma cells based on the expression of canonical markers (Additional file 10: Fig. S4A–D; Additional file 3: Table S3). One of our primary interests was the variation in the composition and functional status of T/NK subsets associated with distinct histologic subtypes. Of the 33,946 T/NK cells obtained after removal of doublets (Methods), 4204 (12.4%) cells were from acinar samples and 18,974 (55.9%) from solid samples. Re-clustering all of these cells revealed ten distinct subsets: four CD4 + T cell subsets (CD4-C1 to C3; CD3D + CD4 +) including one subset of regulatory T cell (Treg; FOXP3 +), three CD8 + T cell subsets (CD8-C1 to C3; CD3D + CD8A +), one cycling T cell subset (Cycling T; MKI67 + TOP2A +), and two subsets of NK cells (NK-C1 and C2, CD3D − TYROBP +) (Fig. 3A–C, and Additional file 10: Fig. S4E–G).

The proportion of the CD8-C1 subset increased incrementally from normal to acinar and solid samples (Fig. 3D). CD8-C1 highly expressed ZNF683 and ITGAE, represented a subpopulation of tissue-resident memory T cells (TRMs), which was reported as a considerable origin of tumor neoantigen specific T cells (Fig. 3B, C) [51, 52]. Besides, the elevated expression of exhaustion-related markers (e.g., LAG3, PDCD1, HAVCR2 and ENTPD1) in CD8-C1 indicated that a considerable fraction of these cells was induced to an exhausted phenotype in the TME (Fig. 3C, Additional file 10: Fig. S4G). Indeed, shared TCRs were frequently observed between ZNF683 + and exhausted T cells in lung tumors [53]. CD8-C2 infiltrated relatively less in tumors, and exhibited an effector memory signature (high GZMK, IFNG and CCL4 but lack of GZMB) and the previously defined “precursor exhausted” T cell signature (high EOMES, NR4A2, and low TCF7) (Fig. 3C, Additional file 10: Fig. S4G) [52, 54]. CD8-C3 was designated as cytotoxic T cells based on their expression of GZMA, GZMB, GNLY and PRF1, and their relative abundance was reduced in tumors. Notably, we observed increased HIF1A expression in the exhausted CD8-C1 subpopulation (Fig. 3C). We further quantified the cytotoxicity, exhaustion, and hypoxia signatures to compare the functional status of CD8 + T cells from different origins (Fig. 3E–G, Additional file 4: Table S4) [25]. The results showed that the exhausted score and hypoxic score increased progressively from normal to acinar and solid samples, while correspondingly, the cytotoxic score of CD8 + T cells from solid samples was lower than that of normal samples but still higher than that of acinar samples. This might suggest that, despite the considerable cytotoxic activity of solid LUAD-derived CD8 + T cells, the intensification of exhaustion induction led to a compromised oncocidal effect, thus contributing to immune escape and poor prognosis of solid tumors. Moreover, multiple immune checkpoint molecules including LAG3, TIGIT and ENTPD1, were significantly upregulated in solid LUAD-derived CD8 + T cells (Fig. 3H, Additional file 4: Table S4). Meanwhile, consistent with our previous finding of a more hypoxic and acidic TME in solid LUADs, the significantly elevated hypoxic score of solid LUAD-derived CD8 + T cells might reflect the involvement of hypoxic mechanism in T cell dysfunction.

GSVA was further conducted to dissect the differences in pathway activities between CD8 + T cells of solid and acinar sample origins (Fig. 3I, Additional file 4: Table S4). The results revealed that cytokine-cytokine receptor interaction was significantly activated in solid LUAD-derived CD8 + T cells. Correspondingly, various cytokines and chemokines, such as CCL4L2, CCL3L1, CCL3, CXCL13 and IFNG, were significantly upregulated in solid LUAD-derived CD8 + T cells (Additional file 10: Fig. S4H, Additional file 4: Table S4H). In particular, multiple metabolic pathways were upregulated in solid LUAD-derived CD8 + T cells (Fig. 3I, Additional file 4: Table S4), some of which were also found to be upregulated in tumor cells from solid samples, such as glycolysis/gluconeogenesis, cysteine and methionine, taurine, hypotaurine, and pyruvate metabolism. This suggested that the metabolic patterns favored by histologic subtypes might have a substantial impact on the metabolism and function of tumor-infiltrating CD8 + T cells. We were gaining the recognition that cellular metabolism constituted an important modulator of T cell replication and function [55]. This prompted us to speculate that solid LUAD-derived T cells were forced to compete for nutrients and oxygen with malignant cells, which could result in metabolic deficiencies and diminished antitumor effects [56]. In comparison, CD8 + T cells derived from acinar samples were significantly enriched in leukocyte transendothelial migration, focal adhesion and T cell receptor signaling pathways (Fig. 3I).

It is widely acknowledged that CD4 + T cells play a prominent role in cancer immunosurveillance and immunotherapy [57]. For the three conventional CD4 + T cell populations, CD4-C1 was identified as effector memory CD4 + T cells based on their expression of ANXA1, CD40LG and GZMA, which infiltrated relatively less in solid samples compared to acinar samples (Fig. 3B–D, Additional file 10: Fig. S4G). Of note, CD4-C2 was designated as a subtype of follicular helper T cells by expressing CXCL13 and TOX2, as well as exhaustion markers including TIGIT, PDCD1, CTLA4 and MAF. This exhausted population, as well, upregulated HIF1A expression and accounted for a relatively higher proportion of solid samples (Fig. 3C, D). CD4-C3 was marked by naïve T cell features such as CCR7, SELL and LEF and was relatively more abundant in solid samples. The infiltration percentage of Treg was elevated in tumors, but comparable in acinar and solid samples (Fig. 3D). The GSVA further revealed that pathways including glycolysis/gluconeogenesis, porphyrin, and chlorophyll metabolism, were significantly upregulated in solid LUAD-derived CD4 + T cells (Additional file 10: Fig. S4I). Dissimilarly, acinar LUAD-derived CD4 + T cells were enriched in natural killer cell mediated cytotoxicity, antigen processing and presentation, and allograft rejection.

To summarize, when compared to acinar LUADs, more T cells in solid LUADs were reprogramed to an exhausted phenotype, and importantly, the anti-tumor effects of T cells in solid LUADs were at least partially circumscribed by the highly nutrient- and oxygen-deprived TME. On the other hand, this also suggested that the therapeutic regimen combining immunotherapy and vascular normalization therapy to improve the TME might be more beneficial for solid LUADs.

### Immunosuppressive phenotype dominates myeloid cells in solid LUADs

**Fig. 4:**
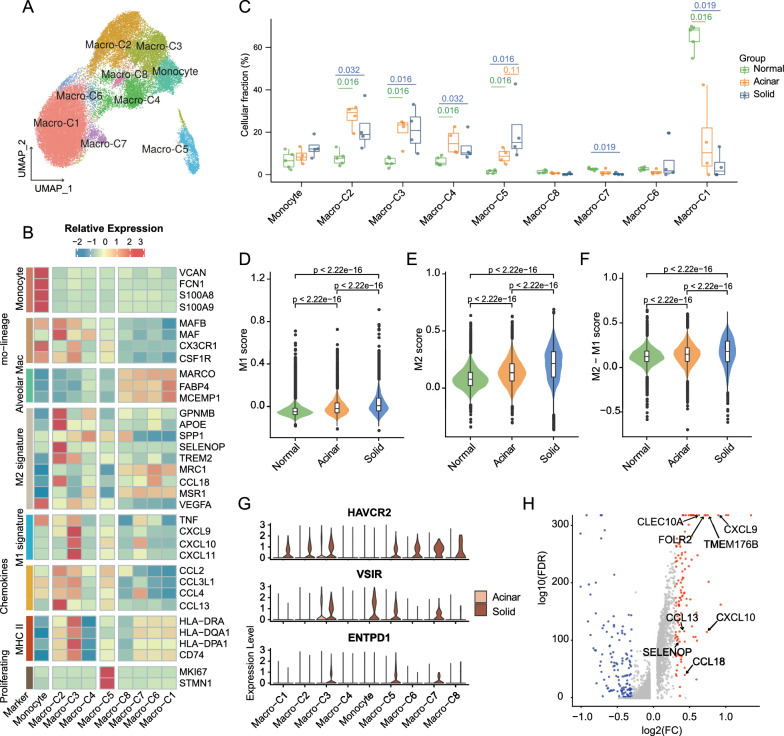
M2 phenotype dominates solid LUAD-derived macrophages. **A**. Cluster-colored UMAP plot of monocytes and macrophages. **B**. Heatmap of normalized expression of monocyte/macrophage marker genes among clusters. **C**. Boxplot showing cellular fractions of each monocyte/macrophage cluster in normal (n = 5), acinar (n = 4) and solid (n = 4) samples. Box centerlines, median; box limits, the 25th and 75th percentiles; box whiskers, 1.5 × the interquartile range. Line segments of different lengths indicate which two groups were compared. Two-sided Wilcoxon rank-sum test. **D-F**. Violin and box plots of M1 **(D)**, M2 **(E)**, and M1-M2 **(F)** signature scores for macrophages in the three subgroups. **G**. Violin plots showing immune checkpoints expression of monocytes/macrophages from solid and acinar samples. **H**. Volcano plot showing the upregulated genes in solid LUAD-derived macrophages

Myeloid cells were further subdivided into monocytes/macrophages and dendritic cells based on the expression of canonical markers (Additional file 11: Fig. S5A–B). Sub-clustering of 36,443 monocytes/macrophages revealed nine subsets, including four subsets of alveolar macrophages (AMs; Macro-C1 and C6-C8: MACRO, FABP4), three subsets of monocyte-derived macrophages (Mo-Macs; Macro-C2 and C3: MAFB, CSF1R; Macro-C4: MAF, SPP1), one subset of actively cycling macrophage (Macro-C5: MKI67, TOP2A) and one subset of monocyte (VCAN, FCN1) (Fig. 4A, B, Additional file 11: Fig. S5C, D). AMs were dominated by the Macro-C1 subpopulation, which was abundant in adjacent normal lungs and had the lowest proportion in solid samples (Fig. 4C). Macro-C6-C8 constituted only a minor fraction of AMs, with Macro-C8 being marked by metallothionein genes, such as MT2A and MT1E, which might be vital in heavy metals binding and processing (Additional file 11: Fig. S5D) [58]. As for the Mo-Macs, the proportions of Macro-C2-C4 were increased in tumors relative to normal samples (Fig. 4C). Macro-C4 was highlighted by high SPP1 expression and absent MHC II expression, which was regarded as a marker of tumor-associated macrophages (TAMs) and was involved in angiogenesis and metastasis promotion (Fig. 4B, C; Additional file 11: Fig. S5D) [59]. Macro-C2 exhibited a conspicuous gene signature of M2 polarization (including MAF, APOE, CCL18 and CCL13), while Macro-C3 was dominated by the M1 signature and highly expressed MHC II molecules (Fig. 4B, Additional file 11: Fig. S5D). Gene ontology annotation of marker genes further revealed that Macro-C2 was associated with neutrophil activation and myeloid leukocyte migration (Additional file 11: Fig. S5E). While Macro-C3 was enriched in antigen processing and presentation, response to IFN-γ and regulation of lymphocyte activation (Additional file 11: Fig. S5F). To compare the general functional characteristics of macrophages between acinar and solid samples, we quantified the phenotypic polarization scores of all macrophages [26]. Intriguingly, we discovered that solid LUAD-derived macrophages had both significantly higher M1 and M2 scores (Fig. 4D, E; Additional file 5: Table S5). Nevertheless, the “M2-M1” score was also significantly higher in solid LUAD-derived macrophages, implying that the immunosuppressive M2 phenotype was predominant in macrophages from solid samples (Fig. 4F). Indeed, we discovered that solid LUAD-derived monocytes/macrophages increased expression of suppressive immune checkpoints including HAVCR2, ENTPD1 and VSIR (Fig. 4G, Additional file 5: Table S5). Beyond that, comparison of gene expression profiling revealed that genes implicated in immunosuppression (FOLR2, TMEM176B, CLEC10A) and chemotaxis (CXCL9, CXCL10, CCL13, CCL18) were upregulated in solid LUAD-derived macrophages (Fig. 4H, Additional file 5: Table S5). FOLR2 encoded the macrophage-specific folate receptor β, which was responsible for folate transportation and was regarded as an attractive therapeutic target for TAMs [60]. The tumor-promoting cytokine CCL18 was also found to be intimately involved in the induction of the M2 phenotype in macrophages [61, 62]. Complementarily, we dissected the functional characteristics of macrophages in bulk data from the TCGA LUAD cohort and obtained consistent results. Those were, signals regulating macrophage and dendritic cell traffic into tumor, as well as enrichment scores for M1 phenotype and myeloid-derived immunosuppression signature increased incrementally with histologic progression (Additional file 11: Fig. S5G–I; Additional file 2: Table S2) [36].

**Fig. 5:**
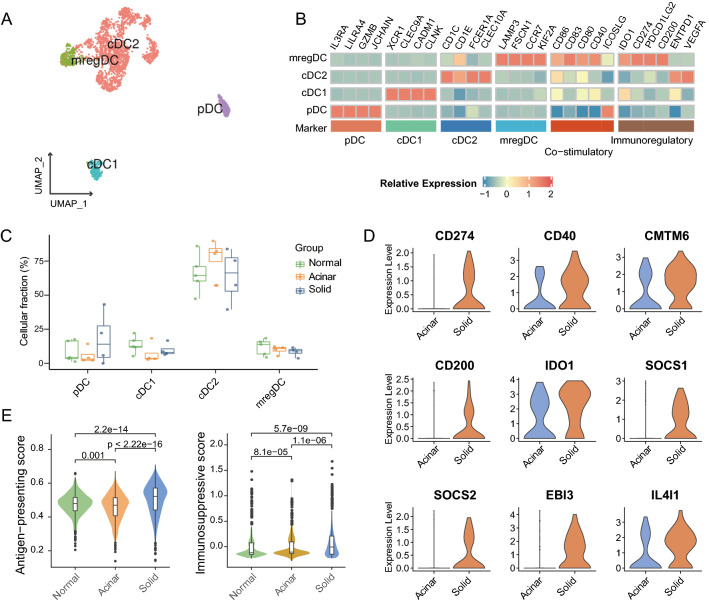
The immunosuppressive phenotype of solid LUAD-derived DCs. **A** UMAP plot colored by DC subtypes. **B** Heatmap of normalized expression of DC marker genes among subtypes. **C**. Boxplot showing cellular fractions of each DC subtype in normal (n = 5), acinar (n = 4) and solid (n = 4) samples. Box centerlines, median; box limits, the 25th and 75th percentiles; box whiskers, 1.5 × the interquartile range. Line segments of different lengths indicate which two groups were compared. Two-sided Wilcoxon rank-sum test. **D**. Violin plots of immunosuppressive molecules expression in mregDC from solid and acinar samples. **E**. Violin and box plots of antigen presentation (left) and immunosuppressive (right) signature scores for DCs in the three subgroups. Comparisons were performed by two-sided Wilcoxon rank-sum test

Dendritic cells are critical in initiating and maintaining anti-tumor T cell immunity. All 2129 dendritic cells were re-clustered and annotated as conventional dendritic cells, type I (cDC1s; CLEC9A); conventional dendritic cells, type II (cDC2s; CD1C); mature regulatory dendritic cells (mregDCs; LAMP3), and plasma dendritic cell (pDCs; LILR4) (Fig. 5A, B). Both cDC1 and pDC were slightly overrepresented in solid samples when compared to acinar samples, and the opposite was the case in cDC2 (Fig. 5C). mregDCs are DC cells in a specific differentiated state associated with tumor antigens, and their immunomodulatory program may be exploited by cancer cells to facilitate immune escape [63]. Although the proportion of mregDCs was comparable between the two subtypes, mregDCs in solid samples were induced to differentiate to a more immunosuppressive phenotype, which corresponded with the overexpression of immunoregulatory genes such as PD-L1, CD200, CMTM6, IDO1, SOCS1, SOCS2, EBI3 and IL4I1 (Fig. 5D, Additional file 5: Table S5). We further quantified the antigen-presentation and the immunosuppressive signatures to ascertain the functional difference between all infiltrating dendritic cells in the three subgroups (Fig. 5E, Additional file 5: Table S5) [27]. Remarkably, solid LUAD-derived DCs have both the highest antigen-presentation score and immunosuppressive score. As previously reported, solid LUADs exhibited a higher rate of somatic mutations, which implies a greater abundance of potential tumor neoantigens [6]. This might partially explain why solid LUAD-derived DCs exhibited increased antigen presentation activity. The tumor microenvironment of solid LUADs, on the other hand, might counteract the anti-tumor effects of DCs by inducing an immunosuppressive phenotype, yet the underlying mechanism of which remained unknown.

Altogether, our results demonstrated that immunosuppressed myeloid phenotypes predominated in solid LUADs, which also suggested that myeloid-directed therapeutic interventions were somehow attractive for counteracting the immunosuppressive TME and eliciting effective antitumor responses in solid LUADs.

### Stromal status disparities in different histologic subtypes

**Fig. 6:**
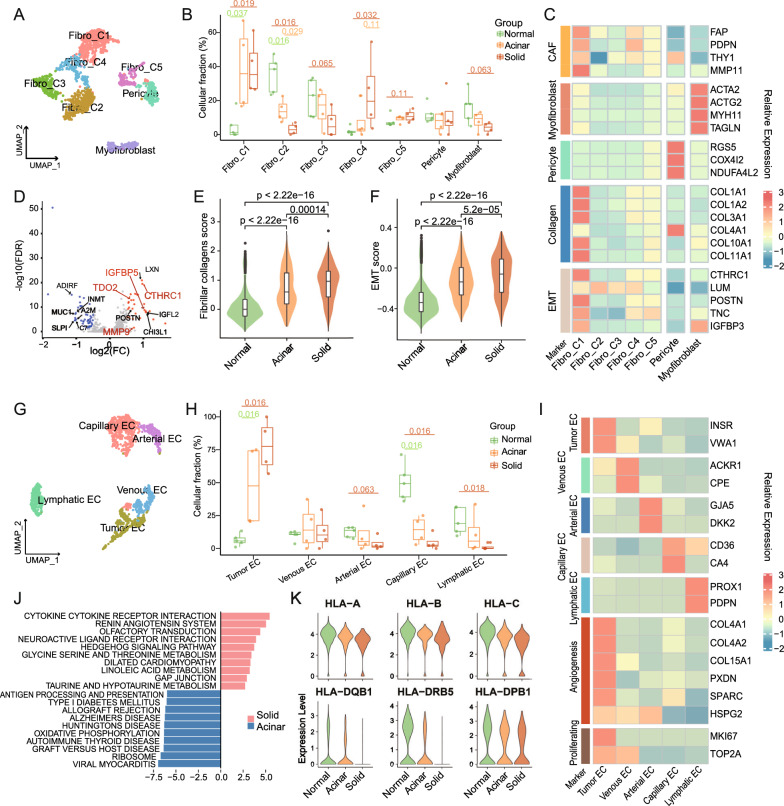
Stromal status disparities in different histologic subtypes. **A**. Cluster-colored UMAP plot of all fibroblasts. **B.** Boxplot showing cellular fractions of each fibroblast cluster in normal (n = 5), acinar (n = 4) and solid (n = 4) samples. Box centerlines, median; box limits, the 25th and 75th percentiles; box whiskers, 1.5 × the interquartile range. Line segments of different lengths indicate which two groups were compared. Two-sided Wilcoxon rank-sum test. **C**. Heatmap of normalized expression of fibroblast marker genes among clusters. **D**. Volcano plot showing the upregulated genes in solid LUAD-derived fibroblasts. **E–F**. Violin and box plots of fibrillar collagens **(E)** and EMT **(F)** signature scores for fibroblasts in the three subgroups. Comparisons were conducted using two-sided Wilcoxon rank-sum test. **G**. Cluster-colored UMAP plot of all endothelial cells. **H**. Boxplot showing cellular fractions of each endothelium cluster in normal (n = 5), acinar (n = 4) and solid (n = 4) samples. Box centerlines, median; box limits, the 25th and 75th percentiles; box whiskers, 1.5 × the interquartile range. Line segments of different lengths indicate which two groups were compared. Two-sided Wilcoxon rank-sum test. **I**. Heatmap of normalized expression of endothelial marker genes among clusters. **J**. Differentially enriched KEGG pathways between tumor endothelial cells from solid and acinar samples revealed by GSVA. **K**. Violin plots showing the expression of selected MHC molecules in endothelial cells

Previous researches support that cancer-associated fibroblasts (CAFs) are implicated in cancer initiation, progression, extracellular matrix (ECM) remodeling, and treatment resistance [64, 65]. Here we addressed the discrepancies between solid LUAD- and acinar LUAD-derived fibroblasts in terms of phenotype constitution and functional status. A total of 1507 fibroblasts were re-clustered and divided into seven subsets including five subsets of fibroblasts (Fibro-C1-C5; COL1A1, COL1A2), one subset of pericyte (RGS5) and one subset of myofibroblast (ACTA2, MYH11) (Fig. 6A–C, Additional file 12: Fig. S6A–C). Acinar and solid samples contributed 585(38.87%) and 329(21.8%) fibroblasts, respectively (Fig. S6A). It was of particular interest that the Fibro-C2 subset, whose relative proportion decreased gradually from normal to acinar and then solid samples, was marked by low expression of fibroblast activation protein (FAP) and specifically overexpressed the transcription factor 21 (TCF21) (Fig. 6B, C, Additional file 12: Fig. S6B, C). Previous study demonstrated that TCF21 was a master regulator of CAF status, and its overexpression diminished the ability of CAF to contract collagen gels, promote tumor growth, invasion and chemotherapy resistance [66]. The Fibro-C1 subset, which was highlighted by classic CAF markers (e.g., FAP, PDPN, MMP11) as well as EMT signature genes (e.g., CTHRC1, POSTN, IGFBP3), was enriched in tumors relative to normal samples (Fig. 6B, C, Additional file 12: Fig. S6C). Simultaneously, Fibro-C1 up-regulated the expression of various collagen fibers (e.g.; COL1A1, COL1A2, COL3A1, COL10A1, COL11A1). This corresponded to the result of gene ontology annotation of the specific markers of Fibro-C1, which revealed that these tumor-enriched fibroblasts were possibly actively participated in the processes of collagen fibril organization and ECM remodeling (Additional file 12: Fig. S6D). Remodeling of the ECM by CAFs might result in a mechanical barrier preventing the migration and infiltration of immune effector cells into the tumor parenchyma [67]. This notion led us to further compare the functional differences of fibroblasts between the two subtypes. Analysis of differentially expressed genes disclosed that CTHRC1 and IGFBP5, both of which played important roles in collagen production and fibrotic progression, were significantly upregulated in solid LUAD-derived fibroblasts (Fig. 6D). In addition, the immunomodulatory gene TDO2 was also found to be upregulated in solid LUAD-derived fibroblasts. We further quantified the fibrillar collagens transcriptional score of all fibroblasts based on the expression of main collagens (i.e., collagens I/ III/V, and fibronectin) that were assembled into large mechanically resilient fibers (Additional file 6: Table S6) [28]. Comparisons between different histologic subgroups demonstrated that the fibrillar collagens transcriptional score was significantly higher in tumor tissues than in adjacent normal lung tissues, and more importantly, higher in solid LUADs than in acinar LUADs, suggesting a more extensive interstitial fibrosis in solid LUADs (Fig. 6E). Moreover, solid LUAD-derived fibroblasts had a significantly higher EMT score (Fig. 6F), which could contribute to the transition of malignant cells from an epithelial phenotype to a more migratory mesenchymal phenotype, thereby promoting distant tumor metastasis and treatment failure. By applying ssGSEA to bulk RNA-seq data from the TCGA LUAD cohort, we subsequently quantified and compared the enrichment scores of fibrillar collagen, matrix remodeling and EMT signatures [36] across histologic subtypes, and the results were consistent with our previous findings (Additional file 12: Fig. S6E–G; Additional file 2: Table S2).

Endothelial cells in the TME not only serve as a physical barrier to tumor cell metastasis but also act as semi-professional antigen-presenting cells involved in the interactions with immune cells (e. g., recruitment and activation of T cells) [68]. Consistent with previous findings, endothelial cells were enriched in normal lungs on account of their hypervascular nature [69]. A total of 1422 endothelial cells, of which 294 (20.7%) and 163 (11.5%) cells were separately from acinar and solid samples, were further re-clustered. The resulting sub-clusters obtained were assigned to known endothelial types in line with the expression of well-established marker genes (Fig. 6G–I and Additional file 13: Fig. S7A–C). When comparing the percentage composition of endothelial categories in different histologic subgroups, we found that tumor endothelial cells (tumor ECs) account for the majority of endothelial population in tumor samples, especially in solid samples. Inversely, lymphatic ECs and capillary ECs were relatively absent in solid samples (Fig. 6H). Notably, tumor ECs were featured by upregulation of angiogenesis-related genes including collagens (e.g., COL4A1, COL4A2), PXDN, SPARC and HSPG2, as well as proliferation markers (e.g., MKI67, TOP2A) (Fig. 6I). These hyperproliferative endothelial cells did not hold the structure and function of normal blood vessels, but might instead exacerbate the hypoxic situation in the microenvironment of solid LUADs [70]. To further elucidate the functional status of tumor endothelial cells, GSVA was conducted and pathway activities were compared between solid and acinar samples. We uncovered that graft versus host disease, allograft rejection, antigen processing and presentation pathways were significantly enriched in acinar LUAD-derived endothelial cells (Fig. 6J). It was documented that increased antigen presentation by endothelial cells assisted in T cell priming and tumor-specific immune activation [71]. Differently, solid LUAD-derived endothelial cells upregulated multiple metabolic pathways such as glycine, serine and threonine metabolism, linoleic acid metabolism. This consisted with previous findings that tumor endothelial cells were transcriptionally reprogrammed to regulate their metabolic functions, which might be associated with the downregulation of their antigen presentation and homing immune cell recruitment functions, and ultimately contributed to tumor escape from immune destruction [71, 72]. In fact, we did find that multiple MHC class I and MHC class II molecules were under-expressed in solid LUAD-derived endothelial cells (Fig. 6K).

Taken together, these encouraging findings enlighten us that therapeutic regimen targeting stromal components may enhance antitumor benefit by facilitating immune-tumor cells interactions in solid LUADs and hold promise to synergize with immunotherapeutic regimens.

## Discussion

Researches dedicated to dissecting the heterogenous TME of distinct histologic patterns of LUADs are well underway. Here we performed a comparative analysis of the TME characteristics between solid and acinar LUAD samples primarily from a single-cell perspective, with the relevant results complemented by bulk transcriptomic and proteomic datasets and validated by immunohistochemistry. Our results suggested that the degree of acidity and hypoxia and the tumor metabolic preferences varied between histologic subtypes and might correspondingly impinge on the metabolism and function of immune components. In addition, ubiquitination modifications might also be involved in the progression of histologic patterns. Indeed, a prevalent state of restrained immune effector function was found in the solid histologic subtype, and discrepancies in stromal cell function could also contribute to the specific immune phenotype of solid LUADs.

Metabolic remodeling fuels tumorigenesis and evolution. However, horizontally, metabolic heterogeneity exits amongst tumors as well as inside an individual tumor, and vertically, adaptive metabolic remodeling may arise along with malignant progression [73]. A concomitant concern is whether the progression of histologic patterns is accompanied by the transformation in metabolic profiles of LUADs. Here we did find a gradient of metabolic alterations and relatively specific metabolic preferences between histologic subtypes, these metabolic properties coincided with the malignant potential of the histologic subtypes and might have a direct or indirect impact on intra-tumoral immune function. Energetically, tumor cells from solid LUADs upregulated glycolytic activity, confronting immune cells, which also relied on glycolysis for effector functions, with a scarcer energy source [56, 74]. On substance metabolism, augmented serine metabolism and pentose phosphate metabolic pathways were observed to supply biomolecules for the exuberant cell replication of solid LUADs. Indeed, we observed a positive correlation between the metabolic activities of tumor glycolytic and pentose phosphate pathway and the proportion of exhausted CD8 + T cells through an independent scRNA-seq dataset of LUAD. Interestingly, unlike the utilization of oxidative phosphorylation by M2 macrophages, the predominant metabolic pattern of M1 macrophages is precisely the glycolytic and pentose phosphate pathway [75]. Furthermore, both hypoxia and higher lactate levels exhibited a facilitative effect on macrophage polarization toward the M2 phenotype. Furthermore, both the hypoxia and lactate levels facilitated the polarization of macrophages toward the M2 phenotype [76]. Hence, oxygen and nutrient deprivation, as well as acidification could be crucial contributors to the predominance of T cell exhaustion and M2 macrophage polarization in solid LUADs. Alongside tumor cells, immunosuppressive cell metabolism was also of concern, an example being the upregulation of IDO1 by solid LUAD-derived mregDCs, which attenuated effector T cell responses by depleting tryptophan and producing the immunosuppressive metabolite kynurenine [76].

Immune checkpoint blocker (ICB) therapies are currently held in high esteem, yet their overall response rate for monotherapy in lung cancer is barely satisfactory [77]. Our study shows that solid LUADs exhibit higher exhausted transcriptional score in T cells relative to acinar LUADs, which raises the question of whether ICB can improve the dysfunctional state of T cells in solid LUADs and enhance their immunosurveillance and immunocidal effects on tumors. It is therefore of great interest to specify the association of histologic composition with the response and the survival benefit of perioperative ICB treatment for LUADs. There is promise in targeting tumor metabolic pathways and moderating metabolic competition in the TME to improve the efficacy of immunotherapy [9, 76]. Study shows that tumors with low glycolysis rates are more sensitive to CTLA-4 blockade, while those tumors with higher glycolytic activities, for example, solid LUADs, may benefit more from the combinational treatment with glycolytic inhibitor and CTLA-4 blockade compared to other histologic subtypes of LUADs [78]. In addition, improved local supply of L-arginine to TME synergistically enhances the efficacy of PD-L1 blockers via a T cell-dependent manner [79]. Remarkably, with respect to other histologic subtypes, we find that folate-mediated one-carbon metabolism and its key gene, MTHFD2, are upregulated in tumor cells from the solid subtype. Intriguingly, the main contributor to the one-carbon unit, serine, is also observed to be metabolically enhanced in tumor cells from solid LUADs [80]. The incrementally increased expression of MTHFD2 with histologic progression is further substantiated at both transcriptional and translation levels by diverse experimental technical scales. MTHFD2, a folate cycling metabolizing enzyme, is considered an attractive metabolic target for tumors by virtue of its multiple roles in metabolic reprogramming and immune regulation [45, 81]. Actually, in the scenario of acute myeloid leukemia, specific MTHFD2 inhibitors potently and selectively repress cancer cell replication while protecting non-neoplastic lymphocytes [82]. To date, specific MTHFD2 inhibitors have shown impressive therapeutic efficacy in preclinical models of diverse cancer types, particularly exhibiting promising antitumor activities in lung adenocarcinoma cell lines, either as a single agent or in synergy with pemetrexed [44, 82–84].Considering the disparities in tumor metabolism and the immune microenvironment in distinct histologic subtypes of LUADs, metabolic targets represented by MTHFD2 hold promise for developing their application in histologic subtype-directed combinatorial immunotherapy. Notwithstanding, more efforts on exploring and validating the efficacy and clinical application protocols of MTHFD2 inhibitors in lung cancer are warranted. We hold out the prospect of adopting MTHFD2 inhibitor as monotherapy or in combination with immunotherapy for the treatment of tumors with elevated MTHFD2 expression, especially for solid LUADs.

Tavernari et al. demonstrated on the spatial scale that regions of solid LUADs exhibited a geographic signature of immune exclusion, i.e., immune infiltration was significantly declined from the immune-enriched tumor margins toward the tumor core, whereas suppressive immune markers such as FOXP3, TIM3 and CTLA4 were distinctively elevated [10]. Notably, the reasons for the spatial distribution and functional differences of immune cells in the solid histologic region remain elusive. Here we propose the following two potential explanations. Firstly, the potential contribution of differences in the spatial distribution of oxygen and nutrients in the tumor regions of solid LUADs; and secondly, the obstruction by ECM components to the migration and movement of immune cells. In the case of the former, we introduce here the tumor model proposed by Lloyd et al. whereby tumor cores tend to maximize their population density and exhibit static, less proliferative phenotypes, while tumor margins are characterized by aggressive proliferative phenotypes [85]. Intriguingly, this model fits highly with the spatial characteristics of the solid pattern of LUAD identified by Tavernari et al. [10]. The harsh metabolic microenvironment created by vicious competition for limited resources in the tumor core may be detrimental to the survival and functional execution of immune cells. Indeed, Lambrechts et al. also suggested that the degree of hypoxia increases progressively from the tumor margin toward the core, whereas most immune cells are inclined to accumulate at the normoxic tumor margin [69]. In the case of the latter, CAFs and their remodeling of the ECM are key factors in structuring the immune infiltration barrier [86]. Based on comparative analysis of transcriptional profiles of the identified fibroblasts, we find that the fibrillar collagen transcriptional level is significantly higher in solid LUAD-derived fibroblasts [28]. And bulk transcriptome-based analysis further confirmed the elevated fibrillar collagen transcription and extracellular matrix remodeling activities in solid LUADs. In addition, it was noteworthy that solid LUADs are often accompanied by substantial intracellular and extracellular mucus production and secretion [1]. This implies that therapeutic regimens targeting CAFs or local ECM potentially promote immune infiltration into the tumor core of solid LUADs, thereby increasing the inter-contact between immune and tumor compartments.

## Conclusions

Collectively, we herein proposed some potential entry points to disrupt the immune exclusion and immunosuppressive phenotype and to potentiate immunotherapeutic efficacy for solid LUADs, yet the realization of these notions requires further investigation and validation at different experimental techniques scales, such as microdissection and spatial omics techniques, as well as tumor models. Furthermore, considering the prospect of possible future applications of histologic subtype-directed LUAD treatment, the development of methods to determine the histologic composition or the presence of certain key components in the tumor prior to treatment is crucial.

## Supplementary Information

**Additional file 1: Table S1.** Characteristics of the LUAD samples included in this study.

**Additional file 2: Table S2.** Data of epithelial cells analysis. (1) Differential expression genes between epithelial cells from solid and acinar samples; (2) Differentially enriched HALLMARK signatures in epithelial cells from solid and acinar samples; (3) Differentially enriched KEGG pathways in epithelial cells from solid and acinar samples; (4) Gene list of signatures for ssGSEA in bulk RNA-seq dataset of the TCGA LUAD cohort.

**Additional file 3: Table S3.** Markers used for differing between primary immune cell types.

**Additional file 4: Table S4.** Data of T/NK cells analysis. (1) Differentially expressed genes between CD8+ T cells from solid and acinar samples; (2) Differentially enriched KEGG pathways between CD8+ T cells from solid and acinar samples; (3) Gene list of signatures for T/NK cells.

**Additional file 5: Table S5.** Data of myeloid cells analysis. (1) Gene list of signatures for macrophages; (2) Differentially expressed genes between macrophages from solid and acinar samples; (3) Gene list of signatures for DCs; (4) Differentially expressed genes between mregDCs from solid and acinar samples.

**Additional file 6: Table S6.** Gene list of signatures for fibroblasts.

**Additional file 7: Fig. S1.** Primary classification of epitheliums, immune cells and stromal cells. **A**–**B**. Representative hematoxylin–eosin (HE) staining images of LUAD manifesting as acinar (**A**) and solid (**B**) growth pattern. The acinar pattern is predominantly glandular, round to oval in shape, with a central lumen surrounded by tumor cells. While the solid pattern consists of cytoplasm-rich polygonal tumor cells forming dense sheets and lacking any other recognizable patterns. The box regions in the upper panel are shown at higher magnification below. Scale bars, 200 μm (top panels) and 50 μm (lower panels). **C**. UMAP plots depicting all cells labeled as epitheliums, immune cells or stromal cells and split by sample types. **D**. UMAP plots of canonical markers for labeling general cell types. E. Heatmaps showing large-scale CNVs for individual epitheliums from tumor samples. Each row represented a cell and the columns represented chromosomal regions. Stromal cells were treated as references (top) and large-scale CNVs were observed in tumor cells (bottom).

**Additional file 8: Fig. S2.** Characteristics of epithelial cells from solid and acinar samples. **A**–**B**. Boxplots showing HIF1A and LDHA mRNA expression across LUAD histologic subtypes in the EAS (**A**) and the CPTAC cohorts (**B**). Box centerlines, median; box limits, the 25th and 75th percentiles; box whiskers, 1.5× the interquartile range. For all comparisons of molecular expression between histologic subtypes, the statistical significance was determined by two-sided Wilcoxon rank-sum test (*P < 0.05, **P < 0.01, ***P < 0.001, ****P < 0.0001, *n.s* not significant). **C**–**D**. UBE2S and UBE2C mRNA expression in the the EAS cohort (**C**), and protein expression in the CPTAC cohort (**D**). Comparisons were performed using two-sided Wilcoxon rank-sum test (*P < 0.05, **P < 0.01, ***P < 0.001, ****P < 0.0001, *n.s* not significant).

**Additional file 9: Fig. S3.** Metabolic differences between epithelial cells from solid and acinar samples. **A**. The average optical density of MTHFD2 immunohistochemical staining in tumor regions from different histologic patterns as semi-quantified by the Image J software. In the box plot, the centre line represents the median, box edges show the 25th and 75th percentiles, and whiskers extend to 1.5× the interquartile range. The statistical significance was determined by two-sided Wilcoxon rank-sum test. **B**. Kaplan–Meier survival curves showing the prognostic difference between the low and high MTHFD2 expression groups in the TCGA LUAD cohort. **C**. Correlation between the expression of MTHFD2 and UBE2S in the TCGA LUAD cohort. P-value was determined by Pearson's correlation test. **D**–**F**. Violin plots showing enrichment scores of one-carbon pool by folate (**D**), pyrimidine metabolism (**E**) and galactose metabolism (**F**) signatures by histologic subtypes in the TCGA LUAD cohort. Global differences were measured by the Kruskal-Wallis test.

**Additional file 10: Fig. S4.** Primary immune cell types identification and T/NK cells analysis. **A**–**C**. UMAP plots of all immune cells colored by major immune types (**A**), sample origins (**B**) and histologic subgroups (**C**). **D**. UMAP plots of selected canonical markers for identifying major immune types. **E**–**F**. UMAP plots of T/NK cells colored by sample origins (**E**) and histologic subgroups (**F**). G. Dot plots showing the top marker genes for each T/NK cell cluster.H. Volcano plot showing differential expression genes between CD8+ T cells from solid and acinar samples. I. Differentially enriched KEGG pathways between CD4+ T cells from solid and acinar samples revealed by GSVA.

**Additional file 11: Fig. S5.** Myeloid cells analysis. **A**. UMAP plot of annotated myeloid cells. **B**. UMAP plots of selected canonical markers for annotating myeloid cells. **C**. UMAP plot of monocytes and macrophages colored by histologic subgroups. **D**. Dot plots showing the top marker genes for each monocyte/macrophage cluster. **E**. Gene ontology annotation of marker genes for the Macro-C2 subset. **F**. Gene ontology annotation of marker genes for the Macro-C3 subset. **G–I. **Violin plots showing enrichment scores of macrophage and DC traffic** (G)**, M1 phenotype **(H)** and immune suppression by myeloid cells **(I)** signatures by histologic subtypes in the TCGA LUAD cohort. Global differences were measured by the Kruskal-Wallis test.

**Additional file 12: Fig. S6.** Fibroblasts analysis. **A**. UMAP plot of fibroblasts colored by histologic subgroups. **B**. UMAP plots showing the expression of selected canonical marker genes for fibroblasts. **C**. Dot plots showing the top marker genes for each fibroblast cluster. **D**. Gene ontology annotation of marker genes for the Fibro-C1 subset. **E**–**G**. Violin plots showing enrichment scores of fibrillar collagens (**E**), matrix remodeling (**F**) and EMT (**G**) signatures by LUAD histologic subtypes in the TCGA cohort. Global differences were measured by the Kruskal-Wallis test.

**Additional file 13: Fig. S7.** Endothelial cells analysis. **A**. UMAP plot of endothelial cells colored by histologic subgroups. **B**. UMAP plots showing the expression of selected canonical marker genes for endothelial cells. **C**. Dot plots showing the top marker genes for each endothelium cluster.

## Data Availability

All data used for this study are publicly published and available with detailed access links described in the **Data resources** section. No new algorithms were developed for this manuscript. All code generated for analysis is available from the authors upon request.

## Abbreviations

LUAD: Lung adenocarcinoma
TME: Tumor microenvironment
TMB: Tumor mutation burden
ICB: Immune checkpoint blocker
EMT: Epithelial-mesenchymal transition
GSVA: Gene set variation analysis
ssGSEA: Single sample gene set enrichment analysis
HIF1A: Hypoxia-inducible factor-1 alpha
LDHA: Lactate dehydrogenase A
TCGA: The cancer genome atlas
EAS: The east Asian ancestry
CPTAC: The clinical proteomic tumor analysis consortium
MTHFD2: Methylenetetrahydrofolate dehydrogenase 2
ROS: Reactive oxygen species
pDCs: Plasmacytoid dendritic cells
DCs: Dendritic cells
cDCs: Conventional dendritic cells
CAFs: Cancer-associated fibroblasts
ECM: Extracellular matrix
MHC: Major histocompatibility complex
FFPE: Formalin-fixed and paraffin-embedded
IHC: Immunohistochemistry
