# Supplementary figures and images for: Comparative profiling of single-cell transcriptome reveals heterogeneity of tumor microenvironment between solid and acinar lung adenocarcinoma
Source: J Transl Med. 2022 Sep 23;20:423. doi: 10.1186/s12967-022-03620-3 (PMC9502652; doi:10.1186/s12967-022-03620-3)

**A**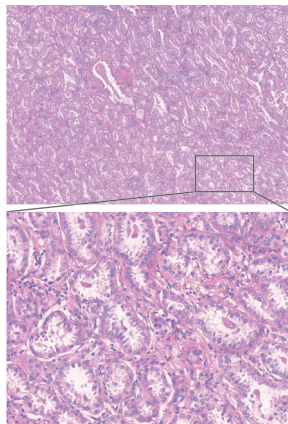**B**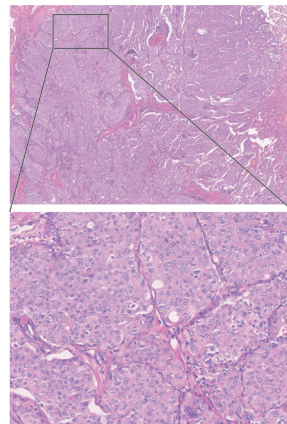**C**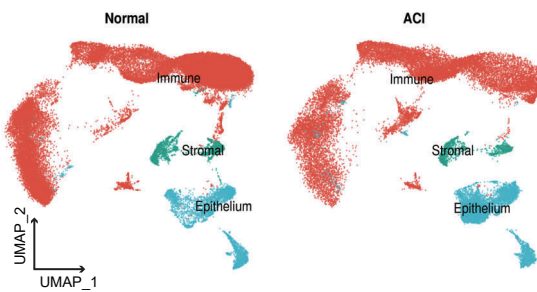**D**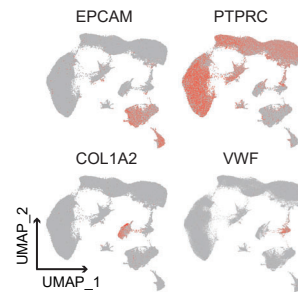

inferCNV

**E**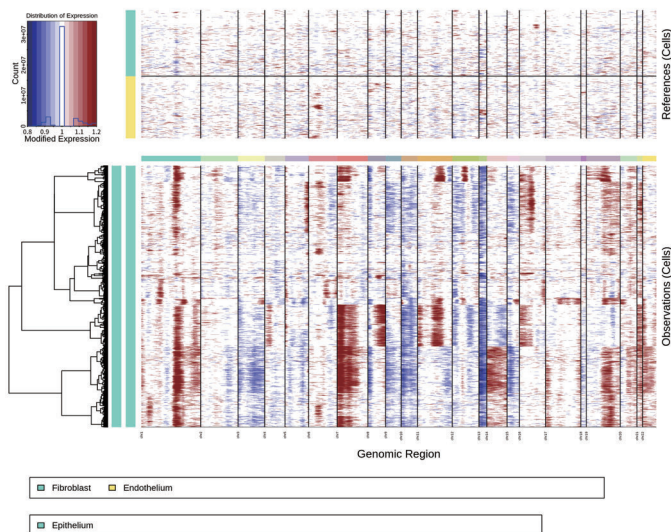

Supplement: Supplementary file 7 — Additional file 7: Fig. S1. Primary classification of epitheliums, immune cells and stromal cells. A–B. Representative hematoxylin–eosin (HE) staining images of LUAD manifesting as acinar (A) and solid (B) growth pattern. The acinar pattern is predominantly glandular, round to oval in shape, with a central lumen surrounded by tumor cells. While the solid pattern consists of cytoplasm-rich polygonal tumor cells forming dense sheets and lacking any other recognizable patterns. The box regions in the upper panel are shown at higher magnification below. Scale bars, 200 μm (top panels) and 50 μm (lower panels). C. UMAP plots depicting all cells labeled as epitheliums, immune cells or stromal cells and split by sample types. D. UMAP plots of canonical markers for labeling general cell types. E. Heatmaps showing large-scale CNVs for individual epitheliums from tumor samples. Each row represented a cell and the columns represented chromosomal regions. Stromal cells were treated as references (top) and large-scale CNVs were observed in tumor cells (bottom). [file 12967_2022_3620_MOESM7_ESM.pdf]

A

EAS

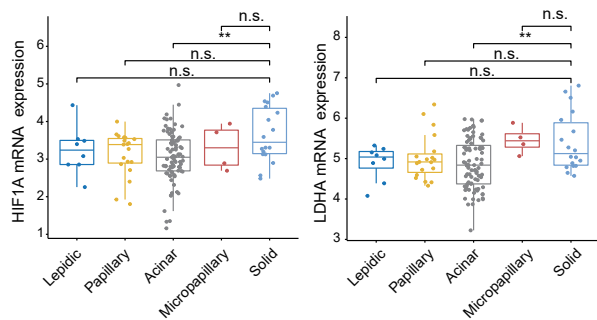

B

CPTAC

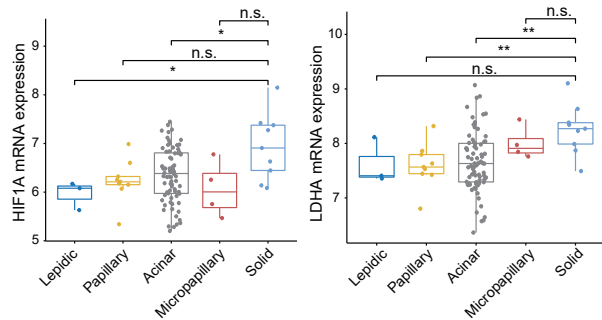

C

EAS

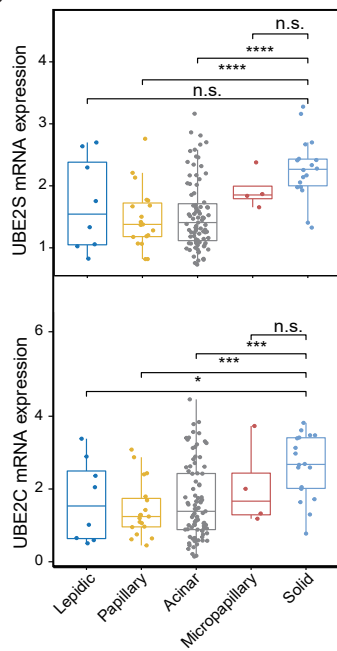

D

CPTAC

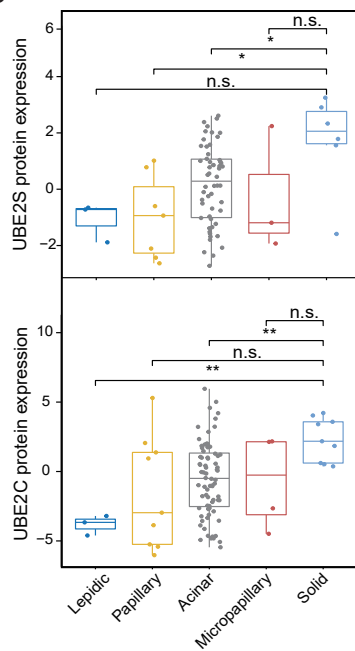

Supplement: Supplementary file 8 — Additional file 8: Fig. S2. Characteristics of epithelial cells from solid and acinar samples. A–B. Boxplots showing HIF1A and LDHA mRNA expression across LUAD histologic subtypes in the EAS (A) and the CPTAC cohorts (B). Box centerlines, median; box limits, the 25th and 75th percentiles; box whiskers, 1.5× the interquartile range. For all comparisons of molecular expression between histologic subtypes, the statistical significance was determined by two-sided Wilcoxon rank-sum test (*P < 0.05, **P < 0.01, ***P < 0.001, ****P < 0.0001, n.s not significant). C–D. UBE2S and UBE2C mRNA expression in the the EAS cohort (C), and protein expression in the CPTAC cohort (D). Comparisons were performed using two-sided Wilcoxon rank-sum test (*P < 0.05, **P < 0.01, ***P < 0.001, ****P < 0.0001, n.s not significant). [file 12967_2022_3620_MOESM8_ESM.pdf]

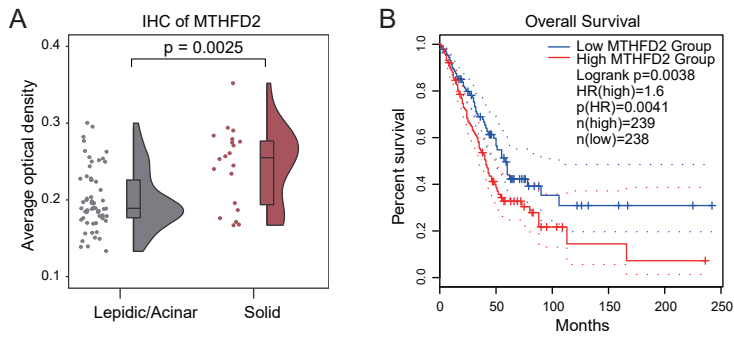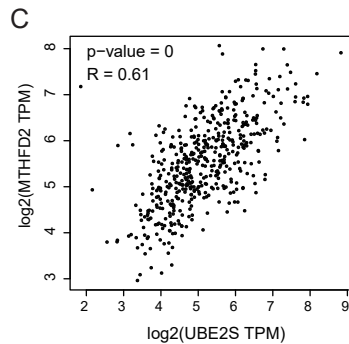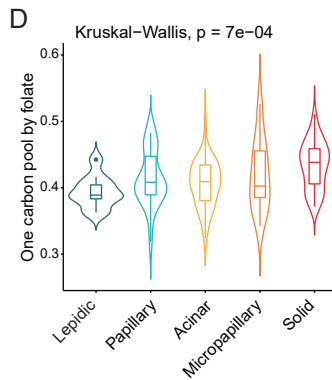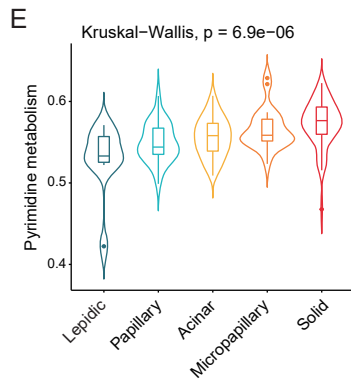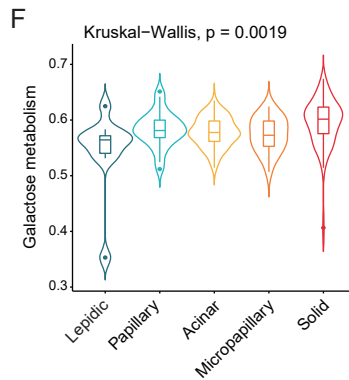

Supplement: Supplementary file 9 — Additional file 9: Fig. S3. Metabolic differences between epithelial cells from solid and acinar samples. A. The average optical density of MTHFD2 immunohistochemical staining in tumor regions from different histologic patterns as semi-quantified by the Image J software. In the box plot, the centre line represents the median, box edges show the 25th and 75th percentiles, and whiskers extend to 1.5× the interquartile range. The statistical significance was determined by two-sided Wilcoxon rank-sum test. B. Kaplan–Meier survival curves showing the prognostic difference between the low and high MTHFD2 expression groups in the TCGA LUAD cohort. C. Correlation between the expression of MTHFD2 and UBE2S in the TCGA LUAD cohort. P-value was determined by Pearson's correlation test. D–F. Violin plots showing enrichment scores of one-carbon pool by folate (D), pyrimidine metabolism (E) and galactose metabolism (F) signatures by histologic subtypes in the TCGA LUAD cohort. Global differences were measured by the Kruskal-Wallis test. [file 12967_2022_3620_MOESM9_ESM.pdf]

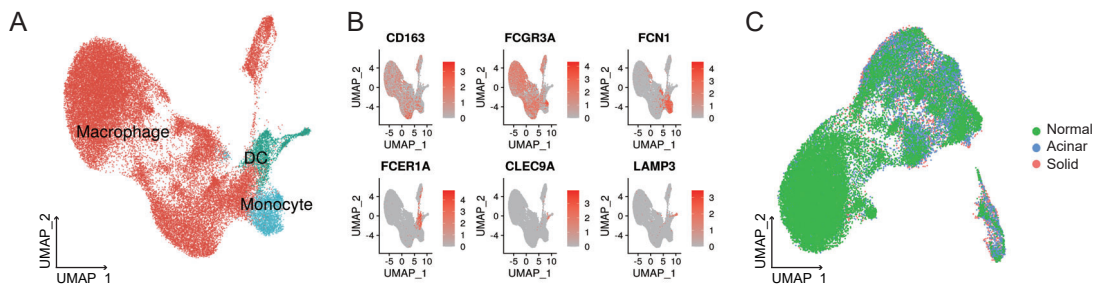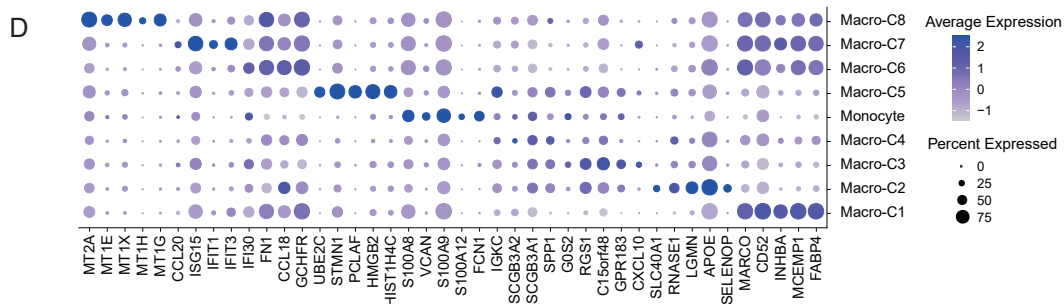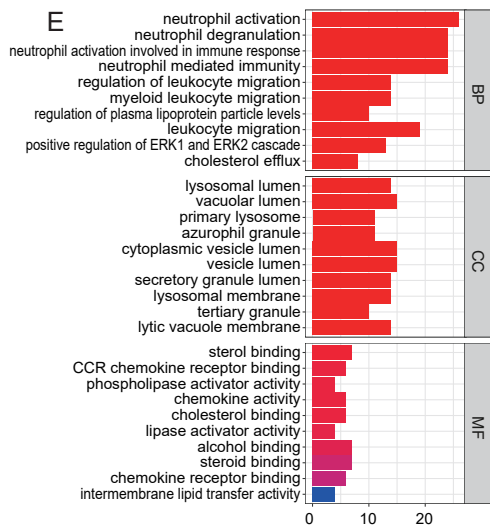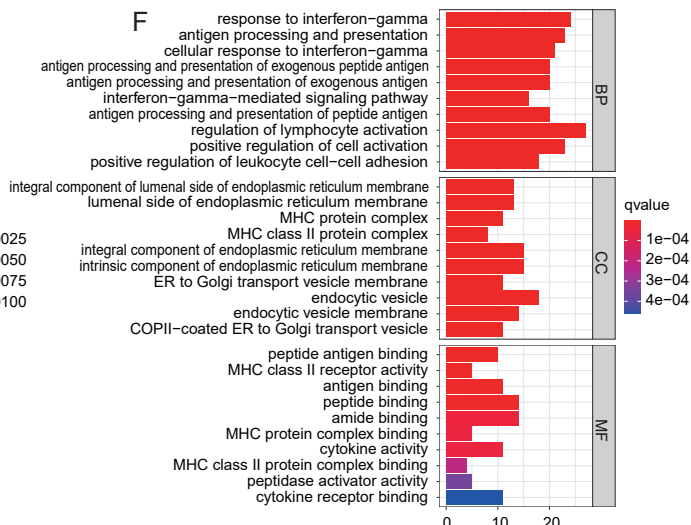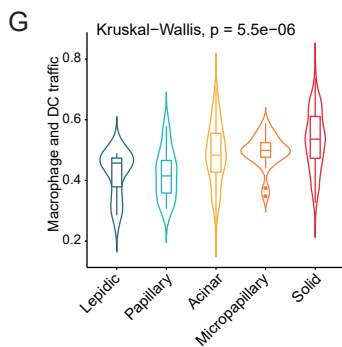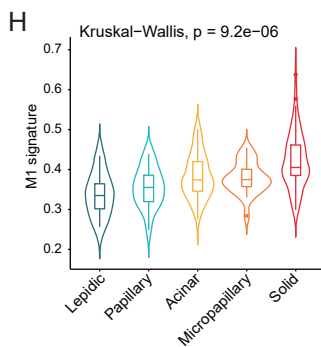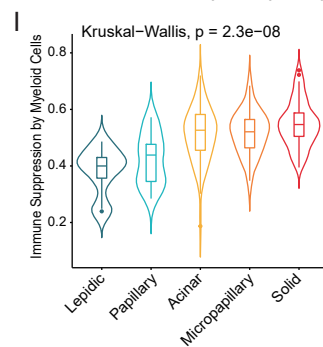

Supplement: Supplementary file 11 — Additional file 11: Fig. S5. Myeloid cells analysis. A. UMAP plot of annotated myeloid cells. B. UMAP plots of selected canonical markers for annotating myeloid cells. C. UMAP plot of monocytes and macrophages colored by histologic subgroups. D. Dot plots showing the top marker genes for each monocyte/macrophage cluster. E. Gene ontology annotation of marker genes for the Macro-C2 subset. F. Gene ontology annotation of marker genes for the Macro-C3 subset. G–I. Violin plots showing enrichment scores of macrophage and DC traffic (G), M1 phenotype (H) and immune suppression by myeloid cells (I) signatures by histologic subtypes in the TCGA LUAD cohort. Global differences were measured by the Kruskal-Wallis test. [file 12967_2022_3620_MOESM11_ESM.pdf]

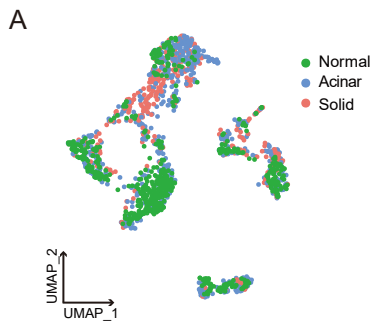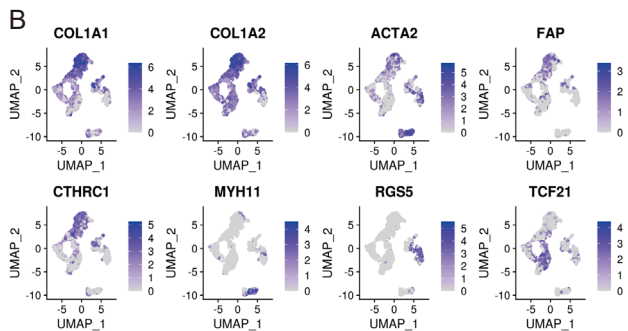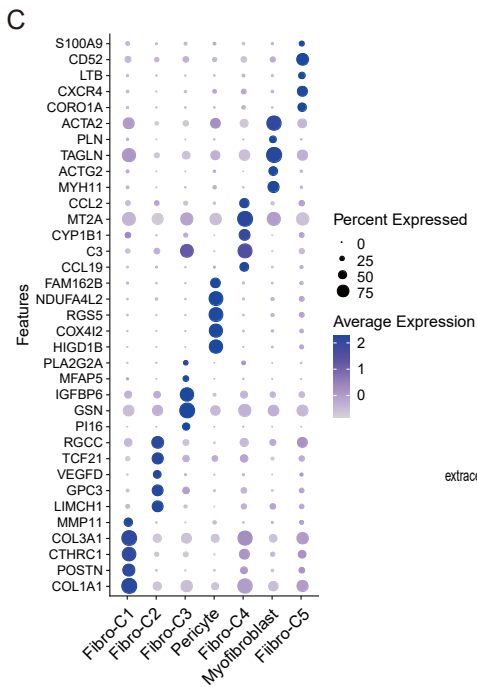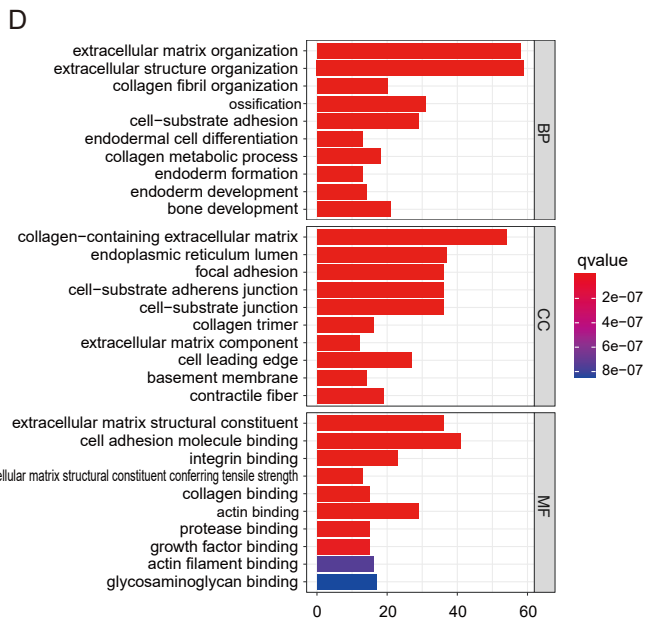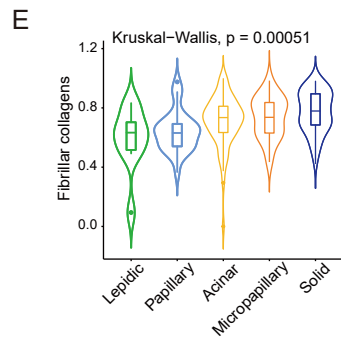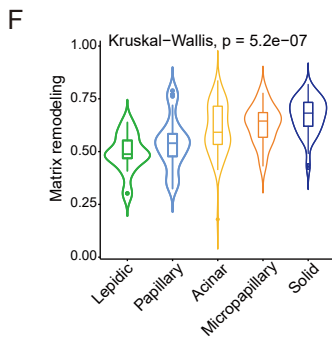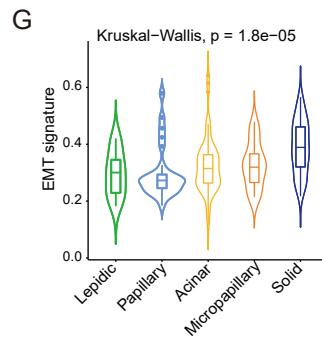

Supplement: Supplementary file 12 — Additional file 12: Fig. S6. Fibroblasts analysis. A. UMAP plot of fibroblasts colored by histologic subgroups. B. UMAP plots showing the expression of selected canonical marker genes for fibroblasts. C. Dot plots showing the top marker genes for each fibroblast cluster. D. Gene ontology annotation of marker genes for the Fibro-C1 subset. E–G. Violin plots showing enrichment scores of fibrillar collagens (E), matrix remodeling (F) and EMT (G) signatures by LUAD histologic subtypes in the TCGA cohort. Global differences were measured by the Kruskal-Wallis test. [file 12967_2022_3620_MOESM12_ESM.pdf]

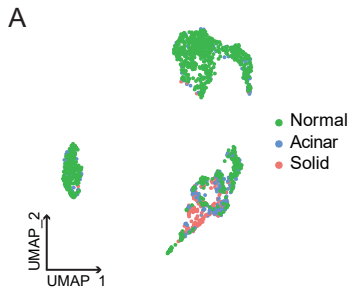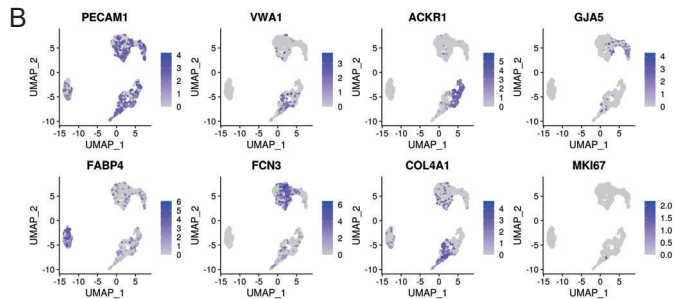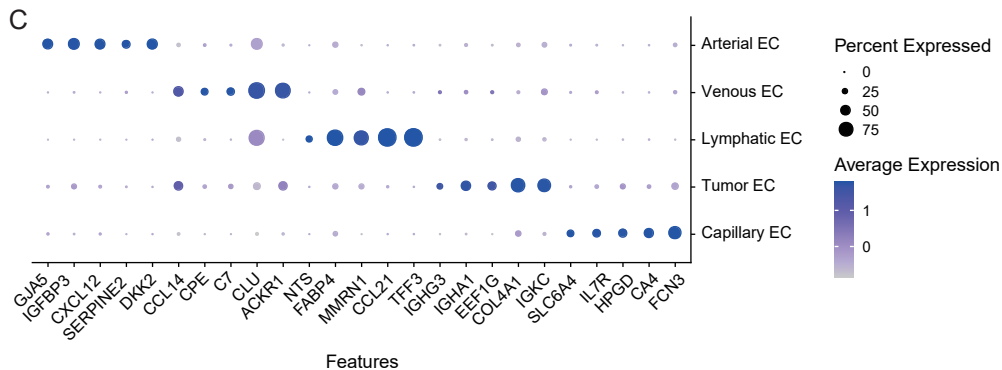

Supplement: Supplementary file 13 — Additional file 13: Fig. S7. Endothelial cells analysis. A. UMAP plot of endothelial cells colored by histologic subgroups. B. UMAP plots showing the expression of selected canonical marker genes for endothelial cells. C. Dot plots showing the top marker genes for each endothelium cluster. [file 12967_2022_3620_MOESM13_ESM.pdf]
