# Supplementary material for: Comparative profiling of single-cell transcriptome reveals heterogeneity of tumor microenvironment between solid and acinar lung adenocarcinoma
Source: J Transl Med. 2022 Sep 23;20:423. doi: 10.1186/s12967-022-03620-3 (PMC9502652; doi:10.1186/s12967-022-03620-3)
Supplement: Supplementary file 10 — Additional file 10: Fig. S4. Primary immune cell types identification and T/NK cells analysis. A–C. UMAP plots of all immune cells colored by major immune types (A), sample origins (B) and histologic subgroups (C). D. UMAP plots of selected canonical markers for identifying major immune types. E–F. UMAP plots of T/NK cells colored by sample origins (E) and histologic subgroups (F). G. Dot plots showing the top marker genes for each T/NK cell cluster.H. Volcano plot showing differential expression genes between CD8+ T cells from solid and acinar samples. I. Differentially enriched KEGG pathways between CD4+ T cells from solid and acinar samples revealed by GSVA. [file 12967_2022_3620_MOESM10_ESM.pdf]

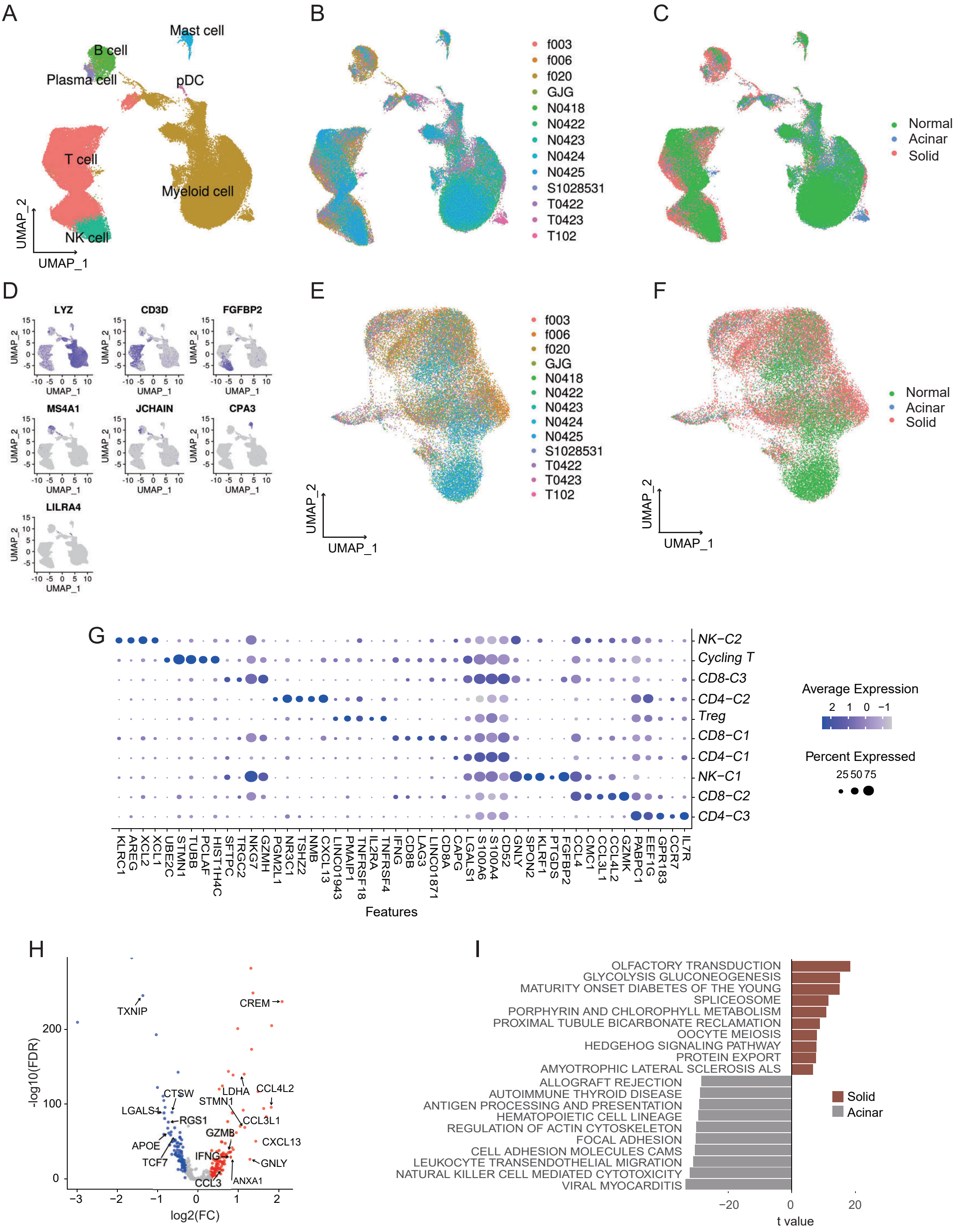
A
Mast cell
B cell
Plasma cell
pDC
T cell
Myeloid cell
NK cell
UMAP_2
UMAP_1
B
f003
f006
f020
GJG
N0418
N0422
N0423
N0424
N0425
S1028531
T0422
T0423
T102
C
Normal
Acinar
Solid
D
LYZ
CD3D
FGFBP2
MS4A1
JCHAIN
CPA3
LILRA4
E
F
G
NK−C2
Cycling T
CD8−C3
CD4−C2
Treg
CD8−C1
CD4−C1
NK−C1
CD8−C2
CD4−C3
Average Expression
2 1 0 −1
Percent Expressed
25 50 75
KLRC1 AREG XCL2 XCL1 UBE2C STMN1 TUBB PCLAF HIST1H4C SFTPC TRGC2 NKG7 GZMH PGM2L1 NR3C1 TSHZ2 NMB CXCL13 LINC01943 PMAIP1 TNFRSF18 IL2RA TNFRSF4 IFNG CD8B LAG3 LINC01871 CD8A CAPG LGALS1 S100A6 S100A4 CD52 GNLY SPON2 KLRF1 PTGDS FGFBP2 CCL4 CMC1 CCL3L1 CCL4L2 GZMK PABPC1 EEF1G GPR183 CCR7 IL7R
Features
H
-log10(FDR)
log2(FC)
TXNIP
CREM
CTSW
LGALS1
RGS1
APOE
TCF7
LDHA
STMN1
CCL4L2
CCL3L1
GZMB
CXCL13
IFNG
GNLY
CCL3
ANXA1
I
OLFACTORY TRANSDUCTION
GLYCOLYSIS GLUCONEOGENESIS
MATURITY ONSET DIABETES OF THE YOUNG
SPLICEOSOME
PORPHYRIN AND CHLOROPHYLL METABOLISM
PROXIMAL TUBULE BICARBONATE RECLAMATION
OOCYTE MEIOSIS
HEDGEHOG SIGNALING PATHWAY
PROTEIN EXPORT
AMYOTROPHIC LATERAL SCLEROSIS ALS
ALLOGRAFT REJECTION
AUTOIMMUNE THYROID DISEASE
ANTIGEN PROCESSING AND PRESENTATION
HEMATOPOIETIC CELL LINEAGE
REGULATION OF ACTIN CYTOSKELETON
FOCAL ADHESION
CELL ADHESION MOLECULES CAMS
LEUKOCYTE TRANSENDOTHELIAL MIGRATION
NATURAL KILLER CELL MEDIATED CYTOTOXICITY
VIRAL MYOCARDITIS
Solid
Acinar
t value
